# The value of a multimodal approach combining radical surgery and intraoperative radiotherapy in the recurrence treatment of gynecological malignancies - analysis of a large patient cohort in a tertiary care center

**DOI:** 10.1186/s13014-024-02537-z

**Published:** 2024-10-25

**Authors:** Tanja Sprave, Raluca Stoian, Natalia Volegova-Neher, Mark Gainey, Michael Kollefrath, Dimos Baltas, Anca-Ligia Grosu, Ingolf Juhasz-Böss, Rieke Schröder, Florin-Andrei Taran

**Affiliations:** 1 Department of Radiation Oncology, University Hospital of Freiburg, Robert-Koch-Strasse 3, 79106 Freiburg, Germany; 2 German Cancer Consortium (DKTK) Partner Site Freiburg, German Cancer Research Center, Heidelberg, Germany; 3 Department of Obstetrics and Gynecology, Medical Center, University of Freiburg, Freiburg, Germany; 4 Faculty of Medicine, University of Freiburg, Freiburg, Germany

**Keywords:** Gynecological cancer, Recurrence, Radiation therapy, Intraoperative radiotherapy, Radical surgery, Electron, High-dose-radiotherapy

## Abstract

**Background:**

Recurrent and locally advanced gynecological malignancies have a poor prognosis. In particularly, pelvic local recurrence after previous radiotherapy and/or positive resection margins during surgical treatment for recurrent disease result in low survival rates. Consequently, locoregional control is of utmost importance in this cohort of patients. The aim of this study was to analyze treatment outcomes and determine prognostic factors for patients treated with surgery and intraoperative radiotherapy (IORT) for recurrent and locally advanced gynecological malignancies.

**Methods:**

40 patients who underwent surgical treatment and IORT between 2010 and 2022 were eligible for inclusion. The median follow-up time was 22 months. The outcomes measured were locoregional control (LRC), overall survival (OS), and survival without distant metastases (DMFS). The Cox proportional hazards model was used for univariate and multivariate analysis to assess the impact of patient variables and treatment factors on the endpoints mentioned. The following variables were analyzed: age at surgical treatment and IORT and initial diagnosis (< 65 vs. ≥65 years, each), disease-free interval (DFI) between initial diagnosis and first recurrence, DFI to surgical treatment and IORT, grading, histology, IORT dose (≤ 13 vs. >13 Gy) and technique (high dose radiotherapy (HDR) vs. IORT using electrons, (IOERT)). Survival curves were generated using the Kaplan-Meier method.

**Results:**

The mean IORT dose was 13.8 Gy (range 10–18 Gy). Cervical carcinoma was most frequently found in 27.5% of patients followed by endometrial carcinoma and vulvar carcinoma in 25% respectively. The final histopathologic results after surgery with IORT showed no residual tumour in 24 patients (60%), microscopic residual disease in 5 patients (12.5%), resection status could not be evaluated in three patients (7.5%) and the resection status was unknown in eight patients (20%). Subsequently, 27.5% of patients also received adjuvant radiotherapy of the local recurrence bed. However, after IORT, 65% of the women suffered a recurrence. Of these, the recurrences were localized: in-field 32.5%, out-of-field 22.5% and margin-of-field 12.5%. The 3- and 5-year OS was 69% and 55% respectively. The 3- and 5-year LRC was 56% respectively. The 3- and 5-year DMFS was 66% and 49%. Whereas the comparison between groups by IORT dose level (≤ 13 vs. >13 Gy) showed a non-significant trend in favor of the higher dose only for OS (*p* = 0.094), but not in LRC and DMFS (*p* > 0.05). OS and DMFS, but not LRC, differed significantly between the HDR-IORT and IOERT groups (*p* = 0.06 and *p* = 0.03,) in favor of the HDR-IORT technique. For HDR-IORT technique a trend towards superior OS and LRC was observed in the univariate analysis: HR 3.76, CI 95%: 0.95–14.881, *p* = 0.059 and HR 2.165 CI 95%: 0.916–5.114, *p* = 0.078

**Conclusions:**

The survival rate for pelvic recurrence in gynecological malignancies remains poor and comparable with historical data from the last two decades. Particularly HDR-IORT, appears to provide a long-term oncological benefit in carefully selected patients.

**Supplementary Information:**

The online version contains supplementary material available at 10.1186/s13014-024-02537-z.

## Introduction

In 2020 gynecological cancers made up roughly 15% of newly diagnosed cancer cases in women as well as 15% of the 4.4 million cancer associated deaths in women worldwide [1].

The 5-year relative survival of gynecological malignancies varies from 81% in the case of uterine cancer to 50% in the case of ovarian cancer [2, 3]. In the case of recurrent disease, the prognosis worsens significantly. Surgical options like pelvic exenteration are limited for central recurrence, radical surgical procedures like laterally extended endopelvic resection are often associated with substantial peri- and postoperative morbidity [4–6]. Furthermore, these operations have a high potential for residual tumour [6, 7].

Patients with recurrent gynecological malignancies and extensive, multimodal treatment history may benefit from the addition of intraoperative radiation therapy (IORT) during radical surgical treatment for recurrent disease [8–11]. However, the use of radiation therapy in this clinical situation is controversial, particularly in patients who have received radiation therapy previously [6]. IORT can enable long-term survival in a carefully selected population of patients with recurrent gynecological malignancy, especially after pre-irradiation in the salvage situation [11]. The possibility of moving healthy surrounding organs out of the pelvis prior to radiation allows for an increase in radiation dose while minimizing the possible damage to surrounding tissue [12–14]. Individualized IORT treatment plans allow for adaption of radiation dose and technique to find the most effective but tolerable local treatment [11].

Previous data from our institution shows that in carefully selected patients, IORT and radical surgery contribute to local control and disease palliation in patients with recurrent gynecological malignancies [15]. Herein we report an update of our single-center experience of using both IORT using electrons (IOERT) and high-dose intraoperative radiotherapy (HDR-IORT) combined with radical surgery as part of a multimodal treatment strategy for locally recurrent gynecological malignancies (LRC) with focus on cervical, endometrial, and vulvar cancer.

## Materials and methods

This retrospective study was conducted at the Medical Center, University of Freiburg, Freiburg, Germany and included patients who were diagnosed with recurrent gynecologic cancer (RGC) and underwent surgical treatment combined with HDR-IORT or IOERT between 2010 and 2022. Medical charts and pathological reports were reviewed.

Institutional criteria for selecting at patients at high risk of recurrence for IORT solely or as an anticipated boost included patients with potentially resectable locally recurrent gynecological cancer (RGC), radical surgery, and negative or microscopic residual tumour on frozen section specimen.

All patients were discussed in the multidisciplinary tumour board. All recurrences were confirmed by biopsy. Before a decision on multimodal treatment was made, preoperative restaging by MRI pelvis and CT of the thorax and abdomen was performed in order to exclude distant metastases. Systemic therapy was applied according to current guidelines and recommendations of the interdisciplinary tumour board.

During surgical resection, the abdomen was fully explored to ensure that there was no evidence of other sites of metastases. Resected surgical specimens were sent for frozen section to confirm margin status. Surgical margins were classified as negative, microscopic, or macroscopic residual tumour. The final decision to administer IORT was made intraoperatively by both the gynecologic oncologist and the radiation oncologist. The IORT was performed using ^192^Iridium microSelectron HDR remote afterloader (Elekta AB, Sweden) technique in a shielded operating room. Due to the complex anatomical surfaces, the Freiburg flap applicator (Nucletron, Veenendaal, The Netherlands) was used in individually tailored sizes for each case. The flexible Freiburg flap consists of the interconnected silicon spheres with a diameter of 1 cm. Thus, the effective distance from the 192Ir HDR source in the catheter tube to the applicator surface is 5 mm. The prescription dose (range 10–18 Gy) was applied to the 5 mm depth from the applicator surface, viz. 10 mm from centre of the 192Ir source.

For easily accessible abdomeno-pelvic localizations with appropriate size, round or oval shape of the recurrence, IOERT using the dedicated linear accelerator (Mobetron, IntraOp Medical, Inc) was selected. The IOERT (range 10–18 Gy) was prescribed to the 90-% isodose. The IOERT energy (range, 6–15 MeV) was chosen to achieve optimal dose coverage for the entire thickness of the tumour cavity sparing the surrounding organs at risk. The size of the IOERT applicator included the tumour bed and a 1–2 cm margin.

Adjuvant EBRT was applied using conventional fractionation (39.6–54 Gy in 22–25 fractions), and if indicated with application of simultaneous integrated boost up to 2.16 Gy. CT-based (Brilliance, CT Big Bore, Philips, Cleveland, OH) three-dimensional treatment planning (Oncentra MasterPlan, Nucletron, Veenendaal, The Netherlands and or Eclipse™ planning systems, Varian Medical Systems, Palo Alto, USA) was performed using individually collimated portals (6 or 18 MV; Synergy; Elekta, Crawley, United Kingdom), intensity-modulated RT (IMRT), or volumetric modulated arc therapy (VMAT) were used to reduce bowel and bladder dose. Since 2019 the EBRT was performed using Surface Guided RT (C-RAD, Catalyst, C-RAD AB, Uppsala, Sweden). The target volume included the surgical cavity of RGC with safety margin, if applicable, taking into account the previous irradiation. In case no RT was performed as part of the initial treatment, pelvic RT was provided in the individual concept analogous to the adjuvant approach for the primary tumour. If required, concomitant with EBRT, cisplatin 40 mg/m2 was administered weekly.

All patients were monitored by gynecologic oncologist and radiation oncologist every three to six months for the first two years, followed by annual visits afterward. Acute postoperative side effects (until 90 days) were evaluated according to the Common Terminology Criteria for Adverse Events version 5.0 (CTCAE v.5). Late toxicity was judged using the modified Late Effects in Normal Tissues criteria (subjective, objective, management, and analytic, LENT-SOMA).

### Statistical analysis

The outcomes measured were locoregional control (LRC), overall survival (OS), and survival without distant metastases (DMFS). All were defined from the date of IORT to the pertinent event. LRC was calculated from the date of resection with IORT to the date of local recurrence. The occurrence of local recurrence was recorded as an event. DMFS was calculated from the date of resection with IORT to the date of progression to other organs. The occurrence of distant metastasis was recorded as an event. Censoring included death from all causes.The Cox proportional hazards model was used for univariate and analysis to assess the impact of patient variables and treatment factors on the endpoints mentioned. The following variables were considered: age at IORT and initial diagnosis (< 65 vs. ≥65 years, each), disease-free interval (DFI) between initial diagnosis and first recurrence (< 12 vs. ≥12 months), DFI to IORT (< 12 vs. ≥12 months), grading, histology, adjuvant EBRT after IORT, IORT dose (≤ 13 vs. >13 Gy) and technique (high dose radiotherapy (HDR) vs. IORT using electrons, (IOERT)). Dates are reported as a mean, median (range) and frequencies. Survival curves were generated using the Kaplan-Meier method. The log-rank test (Mantel-Cox) was used to compare survival curves. *P*-values < 0.05 were considered statistically significant. Statistics were performed with SPSS version 29 (IBM, Armonk, NY, USA).

## Results

A total of 40 women treated by surgery and IORT mean 13.8 Gy (range 10–18 Gy) were identified and included in this analysis. Of which *n* = 21 (52.5%) received HDR - IORT and *n* = 19 (47.5%) IOERT, respectively (Table 2).

The main characteristics and the nature of RGC are summarized in the Table 1. The median age was 58 years (range 26–78). All women had histologically confirmed local RGC, which was most frequently localized in lower pelvis *n* = 16 (45%) and pelvic wall *n* = 11 (27.5%) respectively (Table 1). Cervical carcinoma was the most common diagnosis in eleven patients (27.5%) followed by endometrial carcinoma in ten patients (25%) and vulvar carcinoma in ten patients (25%) each. Accordingly, 50% of the RGC exhibited squamous cell carcinoma. Most women *n* = 25 (62.5%) had the first recurrence, *n* = 8 (20%) the second and in *n* = 7 (17.5%) of the patients indication for treatment was the third or multiple recurrences (Table 1).

**Table 1:** Main characteristics of patients and recurrence

	n (%)
Total patients	40 (100)
Median age (range)	58 (26–78)
*Cancer type*	
Cervical	11 (27,5)
Endometrioidcarcinoma	10 (25)
Vulvar	10 (25)
Ovariancarcinoma	3 (7,5)
Other	6 (15)
*Histology*	
Squamous	20 (50)
Adenocarcinoma	9 (22,5)
Other	11 (27,5)
*Grading bei recurrence*	
G1	1 (2,5)
G2	12 (30)
G3	14 (35)
G4	13 (32,5)
*Number of recurrences*	
1	25 (62,5)
2	8 (20)
3	5 (12,5)
4	1(2,5)
5	1(2,5)
*Localisation of recurrence*	
Pelvic wall	11 (27,5)
Lower pelvic	16 (45)
Para-aortic	2 (5)
Posterior commissure	1(2,5)
Vaginal anterior wall	1(2,5)
Vaginal stump	1(2,5)
Periurethral	2 (5)
Inguinal	2 (5)
Diffuse	2 (5)

Patient, tumour, and recurrence characteristics of patients treated by IORT in our institution between 2010 and 2022 (n = 40)

Abbreviation: *G*: grading, *other*: periurethral, ingulinal, parailiacal, vaginal stump, vaginal anterior wall, posterior commissure, perineal

The final histopathologic results after surgery with IORT showed no residual tumour in 24 patients (60%), microscopic residual disease in 5 patients (12.5%), resection status could not be evaluated in 3 patients (7.5%) and the resection status was unknown in 8 patients (20%) (Table 2). In addition to IORT, 11 women (27.5%) received adjuvant conventionally fractionated EBRT with mean dose of 47.6 Gy (range, 39.6–54), if indicated with simultaneous integrated boost. For these 11 patients, we calculate the cumulative dose resulting from IORT and EBRT for the tumour (EQD2 α/β = 10). Accordingly, they were treated with a mean dose of 74.5 Gy (range 55.6–79.6). Whereas the 29 patients with IORT alone received a mean dose to the tumor (EQD2 α/β = 10) of 31.2 Gy (range 16.6–42). After IORT recurrence occurred in 27 patients (67.5%). Of these, the recurrences were distributed as follows: ‘in field’ in 13 patients (32.5%), ‘out of field’ in nine patients (22.5%) and in ‘field margin’ in five patients (12.5%) respectively (Table 2).

**Table 2:** Treatment characteristics

	n (%)
Total patients	40 (100)
*Primary tumor treatment characteristics*	
*FIGO Stage*	
I/II	14 (35)
III/IV	23 (57,5)
Other	3 (7,5)
Operation	35 (87,5)
Radiotherapy	8 (20)
Radiotherapy + Brachytherapy	15 (37,5)
Brachytherapy	4 (10)
No Radiotherapy/Brachytherapy	13 (32,5)
*Residual tumor after surgery of recurrence*	
R0	24 (60)
R1	5 (12,5)
Rx	3 (7,5)
Unknown	8 (20)
*IORT Dose (Gy) to recurrence*	
Range	10–18
Mean	13.8
HDR - IORT	21 (52,5)
IOERT	19 (47,5)
*Additonal adjuvant EBRT after surgery + IORT*	
yes	11 (27,5)
no	29 (72,5)
EBRT mean dose (range, Gy)	47,6 (39,6–54)
Treatment volume mean (range, ccm)	1159 (129–3557)
Intensity modulated radiotherapy	11 (27,5)
*Recurrence after IORT*	
yes	27 (67,5)
no	13 (32,5)
*Site of recurrence after IORT*	
in field	13 (32,5)
out of field	9 (22,5)
fieldmargin	5 (12,5)

Therapy details for the primary diagnosis of the gynecological carcinoma and for the therapy of local recurrence (n = 40). Staging of primary gynecological cancer was based on the 8th Edition of the UICC TNM classification

Abbreviation: *EBRT*: external beam radiotherapy; *Gy*: Gray; *IOERT*: intraoperative radiotherapy using electrons; *HDR-IORT*: high-dose intraoperative radiotherapy; *IORT*: intraoperative radiotherapy; *NA*: not applicable; *R*: resection status

The median interval between primary tumor resection and surgery with IORT of RGC was 15 months (range 5-112 months). When considering the initial stage of the primary tumour, more than half of the patients *n* = 23 (57.5%) had advanced stage FIGO III-IV disease. The majority of patients *n* = 35 (87.5%) had initial surgical treatment. RT in the adjuvant or primary setting was applied as follows: EBRT in 20%, combined EBRT and brachytherapy in 37.5%, brachytherapy alone in 10%. In one third of the patients (32.5%) no RT was administered in the treatment of the primary cancer (Table 2). 13 (32.5%) patients had no radiotherapy (percutaneous or brachytherapy). The 27 (67,5%) patients were irradiated percutaneously and/or by brachytherapy. Of the 27 pre-irradiated patients: 13 (32.5%) received ‘in field’ RGC, of which two underwent adjuvant radiotherapy, three (7.5%) received ‘out of field’ RGC, of which two underwent adjuvant radiotherapy, and eleven (27.5%) received ‘field margin’ RGC, of which two underwent adjuvant radiotherapy.

The median follow up was 22 months (range 1-154). The 3- and 5-year OS for the whole cohort was 69% and 55% respectively. The 3- and 5-year LRC was 56% respectively. The 3- and 5-year DMFS was 66% and 49%.

Comparisons between women according to age groups (< 65 vs. ≥65 years, each), disease-free interval (DFI) between initial diagnosis and first recurrence (< 12 vs. ≥12 months), DFI to IORT (< 12 vs. ≥12 months), grading, histology, and adjuvant EBRT after IORT resulted in no significant difference in OS, LRC and DMFS (log-rank test, *p* > 0.05 for all) (Additional file 1–3: Figure S1, S2 and S3). Whereas the comparison between groups by IORT dose level (≤ 13 vs. >13 Gy) showed a non-significant trend in favor of the higher dose only for OS (*p* = 0.094, Additional file 1, Figure S1), but not in LRC and DMFS (*p* > 0.05). OS and DMFS, but not LRC, differed significantly between the HDR-IORT and IOERT groups (*p* = 0.06 and *p* = 0.03, Additional file 1–3, Figure S1, S2 and S3) in favor of the HDR-IORT technique.

The univariate analysis with the inclusion of age at IORT and initial diagnosis (< 65 vs. ≥65 years, each), disease-free interval (DFI) between initial diagnosis and first recurrence (< 12 vs. ≥12 months), DFI to IORT (< 12 vs. ≥12 months), grading, histology, adjuvant EBRT after IORT and IORT dose had no significant influence on OS, LRC and DMFS (*p* ≥ 0.05 for all) (Table 3). Of relevance in the univariate analysis, was the non-significant statistical trend towards improved OS and LRC for the HDR-IORT group (OS: HR = 3.76, CI 95%: 0.95–14.881, *p* = 0.059 and LRC: HR = 2.165, CI 95%: 0.916–5.114 *p* = 0.078) (Table 3).

**Table 3:** Univariate analysis using Cox proportional hazard model

	**HR for OS**	**CI 95%**	**p-value**
Age FD ≥ 65	0.558	0,147–2,111	0.39
Age IORT ≥ 65	0.99	0,946–1,035	0.65
DFI between FD and first recurrence	0.995	0,979–1,012	0.584
DFI until IORT	1.001	0,980–1,023	0.894
Grading	1.111	0,599–2,062	0.739
Histology	1.402	0,684–2,873	0.357
IORT-Dose	0.827	0,586–1,168	0.281
HDR-IORT	3.76	0,95 − 14,881	0.059
IOERT	0.266	0,067 − 1,052	0.059
EBRT	1.234	0,36 − 4,223	0.738
	**HR for LRC**	**CI 95%**	**p-value**
Age FD ≥ 65	0.861	0,393–1,886	0.708
Age IORT ≥ 65	0.988	0,958–1,02	0.47
DFI from FD and first recurrence	0.999	0,99 − 1,008	0.818
DFI until IORT	1.002	0,988–1,017	0.761
Grading	1.018	0,679–1,524	0.933
Histology	1.21	0,744–1,967	0.442
IORT-Dose	0.999	0,802–1,244	0.993
HDR-IORT	2.165	0,916–5,114	0.078
IOERT	0.462	0,196–1,091	0.078
EBRT	1.271	0,55 − 2,938	0.575
	**HR for DMFS**	**CI 95%**	**p-value**
Age FD ≥ 65	0.753	0,226–2,511	0.644
Age IORT ≥ 65	1.003	0,96 − 1,048	0.894
DFI from FD and first recurrence	0.999	0,986–1,013	0.931
DFI until IORT	0.995	0,972–1,018	0.646
Grading	1.078	0,606–1,917	0.799
Histology	1.194	0,608–2,345	0.607
IORT-Dose	0.885	0,659–1,188	0.417
HDR-IORT	2.391	0,706–8,102	0.161
IOERT	0.418	0,123–1,417	0.161
EBRT	2.412	0,775–7,507	0.128

Abbreviations: *CI* confidence intervall; *FD* first diagnose; *DFI* disease free intervall; *HDR* high dose radiotherapie; *IOERT* intraoperative electrons radiotherapy; *EBRT* external beam radiotherapy

Table 4 displays the toxicity profile of the study population. No patient experienced a higher grade ≥ 4 event. In the pre-irradiated patients with ‘in field’ RGC, who had received adjuvant EBRT after surgery and IORT, a one grade 2 lymphoedema of the lower extremity and one grade 3 vesicovaginal fistula were observed regarding acute toxicity, respectively (Table 4). Grade 1 chronic recurrent cystitis was observed in a pre-irradiated patient with ‘in field’ RGC who had received adjuvant EBRT after surgery and IORT. In a pre-irradiated patient with ‘field margin’ RGC, who had received adjuvant EBRT after surgery and IORT, a grade 3 late toxicity infected urinoma was observed.

**Table 4:** Toxicity

Total patients 40 (100)	n (%)		
	Grade 1	Grade 2	Grade 3
*Acute toxicity after surgery and IORT*			
Lymphedema		1(2.5)	
Bleeding			1(2.5)
Wound infection			1(2.5)
Wound retention	3(7.5)		
Enterocutaneous fistula		1(2.5)	
Vesicovaginal fistula			2(5.0)
*Late toxicity*			
Lymphedema		3(7.5)	
Cystitis	1(2.5)		
Wound healing disorder			2(5.0)
Skin induration	1(2.5)		
Infected urinoma			1(2.5)
Vesicovaginal fistula		2(5.0)	

Acute and chronic radiotherapy-related toxicities after HDR-IORT according to the Common Terminology Criteria for Adverse Events (CTCAE v5.0) and the modified Late Effects in Normal Tissues criteria (subjective, objective, management, and analytic, LENT-SOMA)

In the entire cohort, acute wound retention disorders grade 1 were most frequently reported in three women (7.5%). Acute grade 3 events such a bleeding (2.5%), wound infection (2.5%), and two vesicovaginal fistulas (5%) were also recorded. The most common late adverse events in the entire cohort were grade 2 lymphedema of the leg (7.5%) and grade 3 vesicovaginal fistula (5%) (Table 4).

## Discussion

**Fig. 1:**
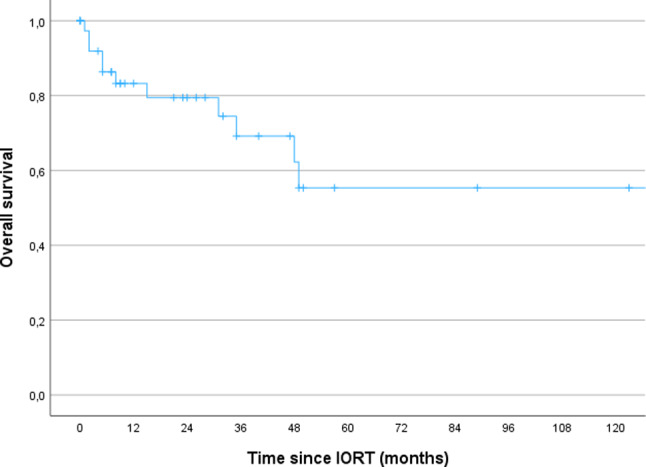
Kaplan-Meier curves regarding OS

**Fig. 2:**
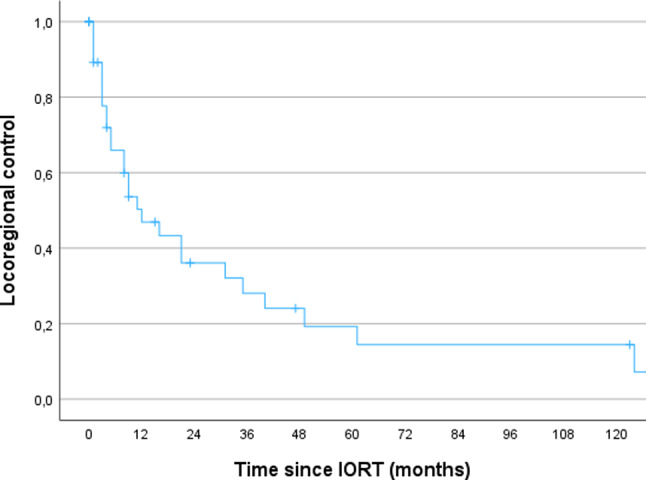
Kaplan-Meier curves regarding LRC

**Fig. 3:**
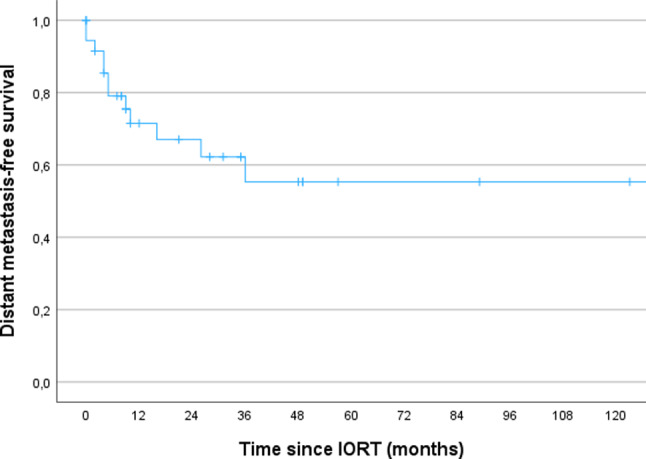
Kaplan-Meier curves regarding DMFS

In this report, we re-evaluated our experience with IORT in combination with radical surgery as part of a multimodality treatment strategy for RGC. Our results demonstrated that the multimodal approach is well tolerated but still associated with poor survival. After a median follow-up of 22 months, the 5-year OS was 55%, the LRC was 56% and the DMFS was 49% (Figs. 1, 2 and 3). Jablonska et al. reported the similar results with a 14-year local control rate of 51%, DFS and OS rates of 15–20% [16]. A phase II study on perioperative HDR reported high local control at around 80% and comparable OS at 46% after 16 years in non-pre-irradiated patients [17]. In addition, the authors observed a local control rate of approx. 60% and an OS rate of 16% in ten pre-irradiated patients after 14 years [17]. In our cohort, 67% of the patients were pre-irradiated and showed a comparable LRC albeit after a short follow-up (Table 2; Fig. 2).

IORT as single-dose can achieve promising local control after optimal surgery and complete resection [6, 10, 18]. In our cohort, the final histopathologic findings detected in 24 patients (60%) no residual tumour, microscopic residual disease in five patients (12.5%), and unfortunately in eleven patients (27.5%) no information on resection status was available (Table 2). For this reason, we did not include the incompletely reported resection status in our univariate analysis and comparison of the survival curves.

Conversely, IORT alone cannot improve OS and local recurrence rates in an unfavourable constellation with need for extensive surgery, residual tumour, and pre-radiation [19]. In addition to IORT, postoperative EBRT can achieve a higher cumulative radiation dose locally and locoregionally and could have an oncological advantage [20], especially in patients who have not been previously irradiated. Sole et al. reported that in lymph node recurrence, the addition of EBRT to IORT and surgery can improve local control without worsening the acute and chronic toxicity profile [21]. Importantly, this combination of EBRT and IORT in lymph node recurrence showed a significant benefit in local control (HR = 4.11, *p* = 0.04) and disease-free survival (HR 2.76, *p* = 0.04) [21]. Furthermore, Sole et al. found that EBRT for the primary tumour region provided no significant benefit in this setting. Consistently, the combination of IOERT and perioperative EBRT resulted in an improvement in locoregional control, especially in the case of tumour fragmentation with R0 resection [22]. In our study group, only eleven (27.5%) patients had received adjuvant EBRT with a median dose of 47.6 (39.6–54) Gy (Table 2), but with no significant difference to the cohort without adjuvant EBRT (Table 3). Notably, in our non-pre-irradiated cohort, a curative dose of adjuvant EBRT of 47.6 (39.6–54) Gy was used, which is particularly relevant for the treatment of subclinical tumour residuals. However, in the case of full-dose pelvic pre-irradiation, Backes et al. used postoperative EBRT with a mean of 26 Gy (range 10 to 40 Gy) in addition to IORT, which is probably an insufficient dose for tumour control [19]. In fact, the negative result of Backes et al. is similar with our findings regarding adjuvant EBRT [19], but the study populations and the prescribed doses are not comparable. It is conceivable, that the proportion of patients with IORT and adjuvant EBRT in our study (27.5%) is too small and the follow-up too short to show a significant oncological benefit. The development of radioresistance in gynecological tumours is based on complex interactions [23]. The emergence of RGC is probably associated with varying degrees of radioresistance. Consequently, radioresistance may explain the inconsistency of the perioperative EBRT impact. Congruent to the above-mentioned studies, we did not observe any grade 4 toxicities (Table 4). Furthermore, in our cohort the pre-irradiated patients who also received adjuvant EBRT did not suffer from increased toxicity rates. In our study for EBRT, intensity modulated radiotherapy (IMRT) and daily image guidance were routinely used for precise and conformal delivery. IMRT reduces side effects by better sparing the surrounding organs at risk compared to conventional 3D-RT with comparable oncological outcome [24, 25]. This may explain the mild toxicity profile in our study compared to older studies prior the widespread adoption of intensity modulated RT in the community.

Particularly in the presence of local recurrence after prior radiotherapy, HDR-IORT offers local dose escalation in the tumour bed with simultaneous toxicity reduction due to the steep dose decrease in the surrounding organs at risk. Furthermore, the application of HDR-IORT enables optimal dose coverage both in the anatomically difficult to access concave and large tumour cavities (> 10 cm). In addition, the HDR-IORT dose is prescribed to the 5 mm depth from the applicator surface. This leads to a maximum dose of up to approx. 150% directly at the centre of the contact surface between the applicator and the tumour cavity. This can lead to localized necrosis and potential increased toxicity. In our cohort, univariate analysis revealed a non-significant trend in OS and LRC with (OS: HR = 3.76, CI 95%: 0.95–14.881, *p* = 0.059 and LRC: HR = 2.165, CI 95%: 0.916–5.114 *p* = 0.078) in favor of the HDR-IORT technique was observed (Table 3).

19 women (47.5%) in our cohort were treated with IOERT (Table 2). IOERT was applied to the round-oval tumour cavities in the easily accessible areas. Furthermore, the IOERT energy (range 6–15 MeV) was selected for optimal dose coverage based on the preoperative diagnosis, intraoperative situs and resection margins. Remarkably, the application of HDR-IORT in our study showed a significant benefit for OS and DMFS (*p* = 0.06 and *p* = 0.03, Additional file 1–3, Figure S1, S2 and S3).

The addition of new agents to the standard chemotherapy regimen provides promising results and underlines the individualized therapeutic approach. The phase III study showed that the combination of dostarlimab and carboplatin-paclitaxel significantly prolonged progression-free survival in locally advanced or recurrent endometrial cancer [26]. Progression-free survival at 24 months was 36.1% (95% CI, 29.3–42.9) in the dostarlimab cohort vs. 18.1% (95% CI, 13.0-23.9) the placebo cohort (hazard ratio, 0.64; 95% CI, 0.51 to 0.80; *P* < 0.001) [26]. Patients with mismatch repair-deficient and, microsatellite instability-high tumours had the greatest progression-free survival benefit. Remarkably, the proportion of included patients in the recurrence situation was approximately 50% [26]. In addition, dostarlimab has a robust antitumour effect with a moderate toxicity profile [27], which is a key prerequisite for patient compliance. A potential synergistic effect of combined IORT and immunotherapy should be evaluated prospectively.

Despite these concordant results, the limitations of this analysis should be noted. This retrospective study was conducted in only one institution and included a heterogeneous cohort with individualized treatment approaches. The choice of IORT dose was predominantly based on intraoperative positive frozen margins and sparing of surrounding organs. This may have led to undertreatment in the critical regions and contributed to in-field recurrence in one third of the women (Table 2). Furthermore, the development of field margin recurrence was observed in five women (12.5%) (Table 2), possibly due to underestimation of the actual extent of recurrence. As highlighted above, the choice of the appropriate applicator size for IOERT and flab size for HDR-IORT may have been incorrectly small. As a result, the cavity margins did not receive a sufficient dose for durable tumour control. In addition, only 17 patients (27.5%) were irradiated percutaneously with different doses. Therefore, in our small cohort the actual benefit of additional percutaneous dose saturation in terms of improved local control cannot be conclusively answered. This in turn limits the transferability to other patient groups outside our institution. The short follow-up in our cohort provides only limited evidence of late toxicity and oncological survival benefit occurring over a longer period.

## Electronic supplementary material

Below is the link to the electronic supplementary material.

Supplementary Material 1

Supplementary Material 2

Supplementary Material 3

## Data Availability

No datasets were generated or analysed during the current study.

## Abbreviations

DFI: Disease free interval
DMFS: Distant metastases free survival
EBRT: External beam radiotherapy
Gy: Gray
IMRT: Intensity modulated radiotherapy
IORT: Intraoperative radiotherapy
IOERT: Intraoperative radiotherapy using electrons
LRC: Locoregional control
MeV: Mega electron volt
OS: Overall survival
RGC: Locally recurrent gynecological cancer
RT: Radiotherapy
VMAT: Volumetric modulated arc therapy
EQD2: Equivalent Dose in 2 Gy fractions
