# Supplementary material for: The value of a multimodal approach combining radical surgery and intraoperative radiotherapy in the recurrence treatment of gynecological malignancies - analysis of a large patient cohort in a tertiary care center
Source: Radiat Oncol. 2024 Oct 25;19:147. doi: 10.1186/s13014-024-02537-z (PMC11515090; doi:10.1186/s13014-024-02537-z)
Supplement: Supplementary file 1 — Supplementary Material 1 [file 13014_2024_2537_MOESM1_ESM.docx]

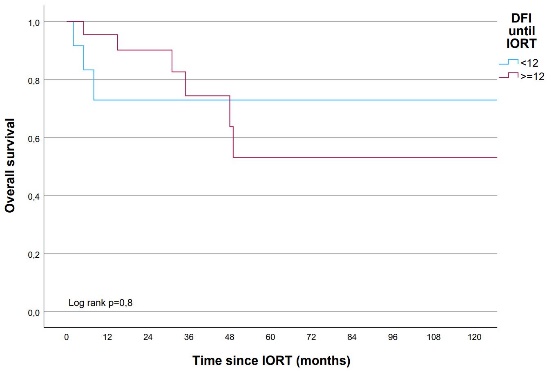


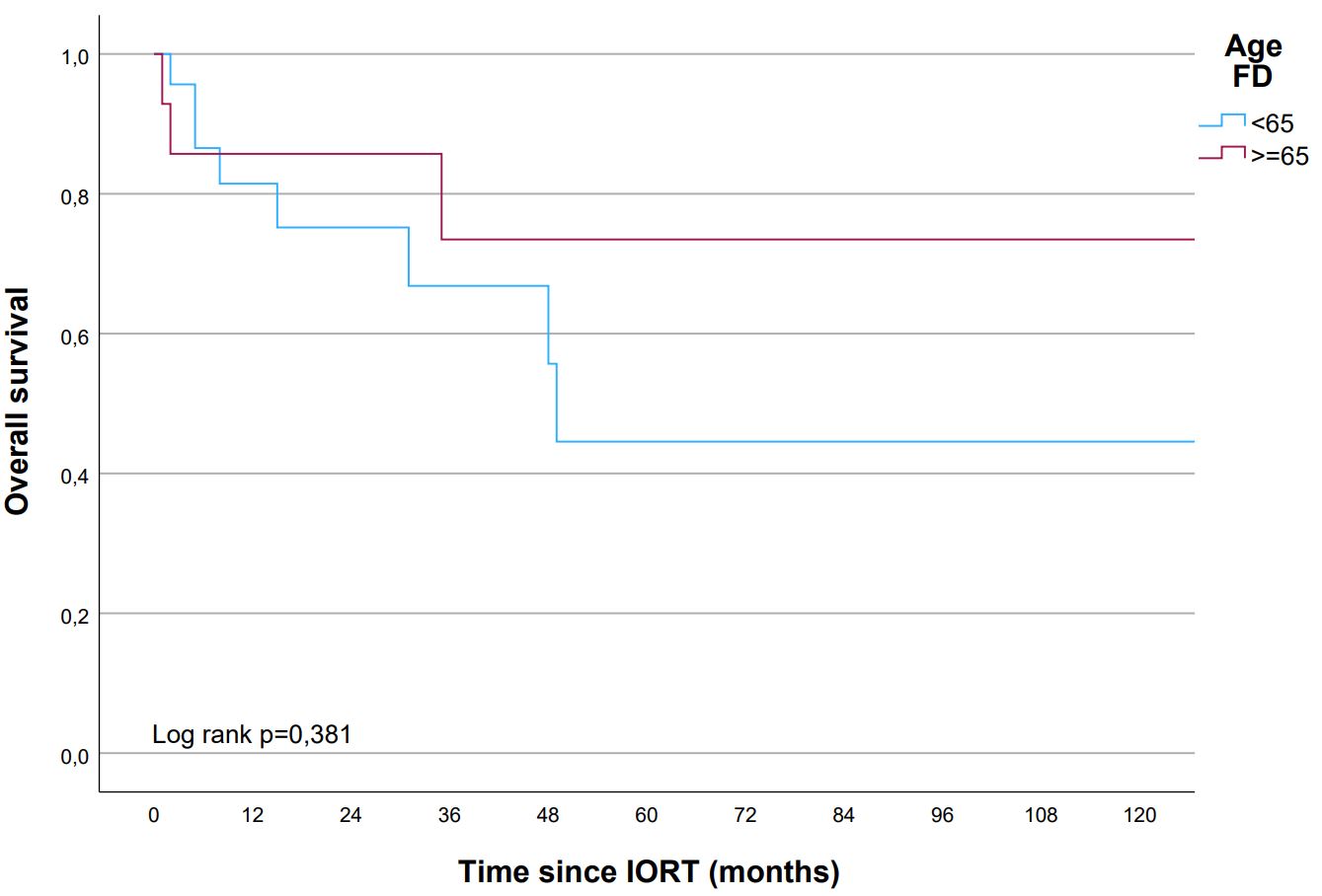

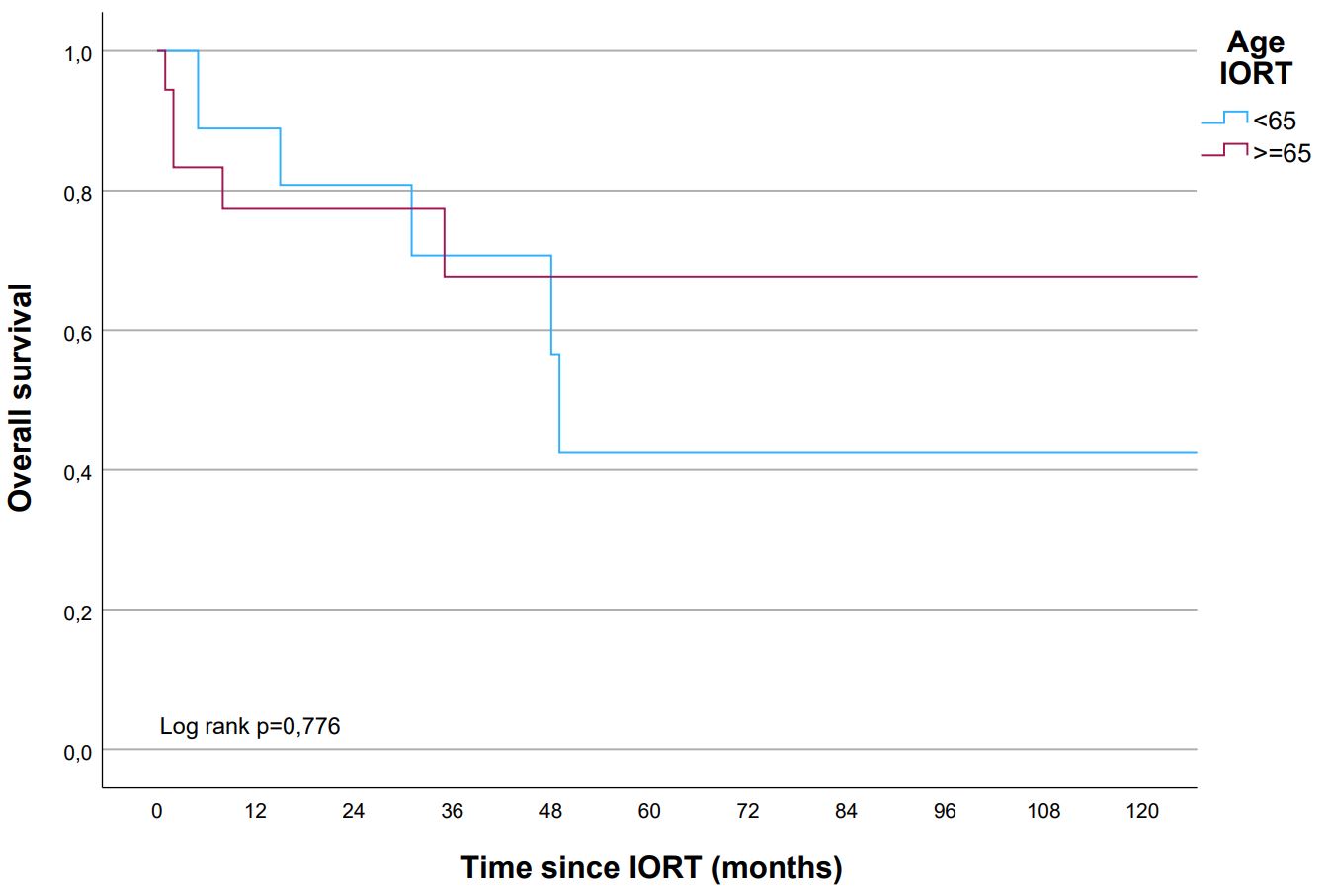

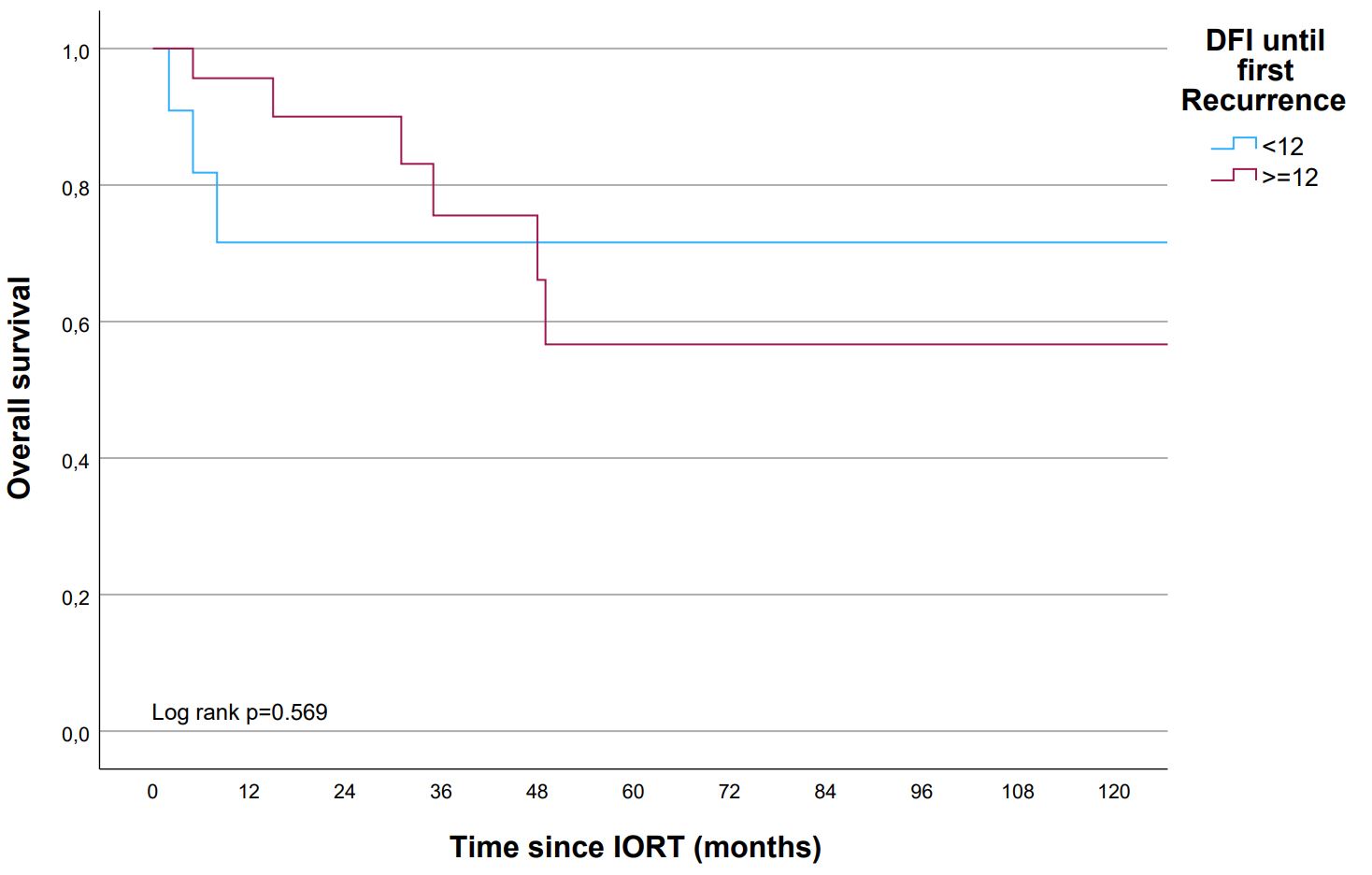


No at risk No at risk No at risk No at risk

<65 years 26 13 10 6 6 3 3 3 2 2 2 <65 years 22 11 8 5 5 2 2 2 1 1 1 <12 months 13 6 4 3 2 2 2 2 2 2 2 <12 months 13 6 4 3 2 2 2 2 2 2 2

≥65 years 14 10 9 6 4 2 2 2 2 2 2 ≥65 years 18 12 11 7 5 3 3 3 3 3 3 ≥12 months 24 17 15 9 8 3 3 3 2 2 2 ≥12 months 24 17 15 9 8 3 3 3 2 2 2


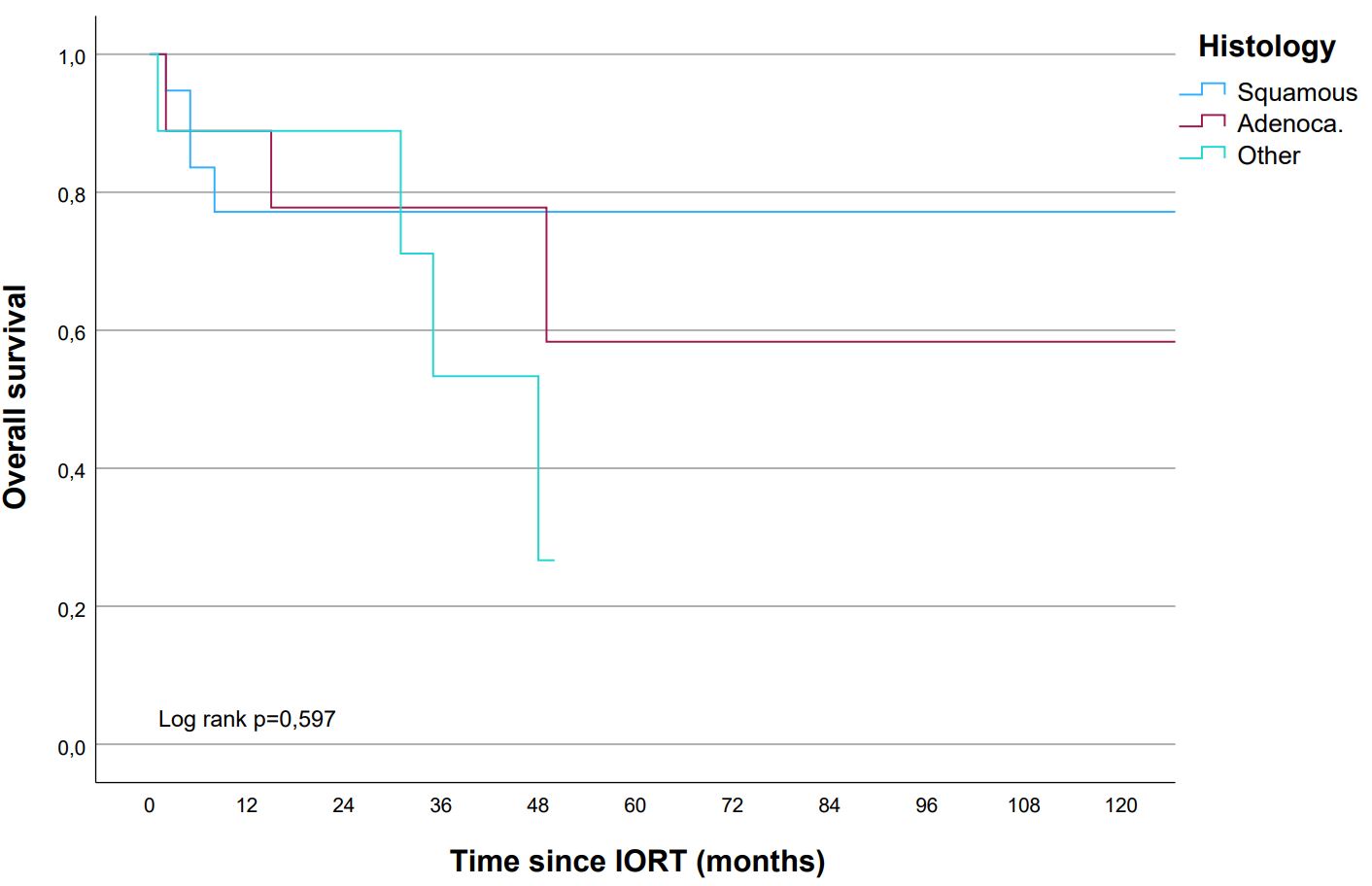

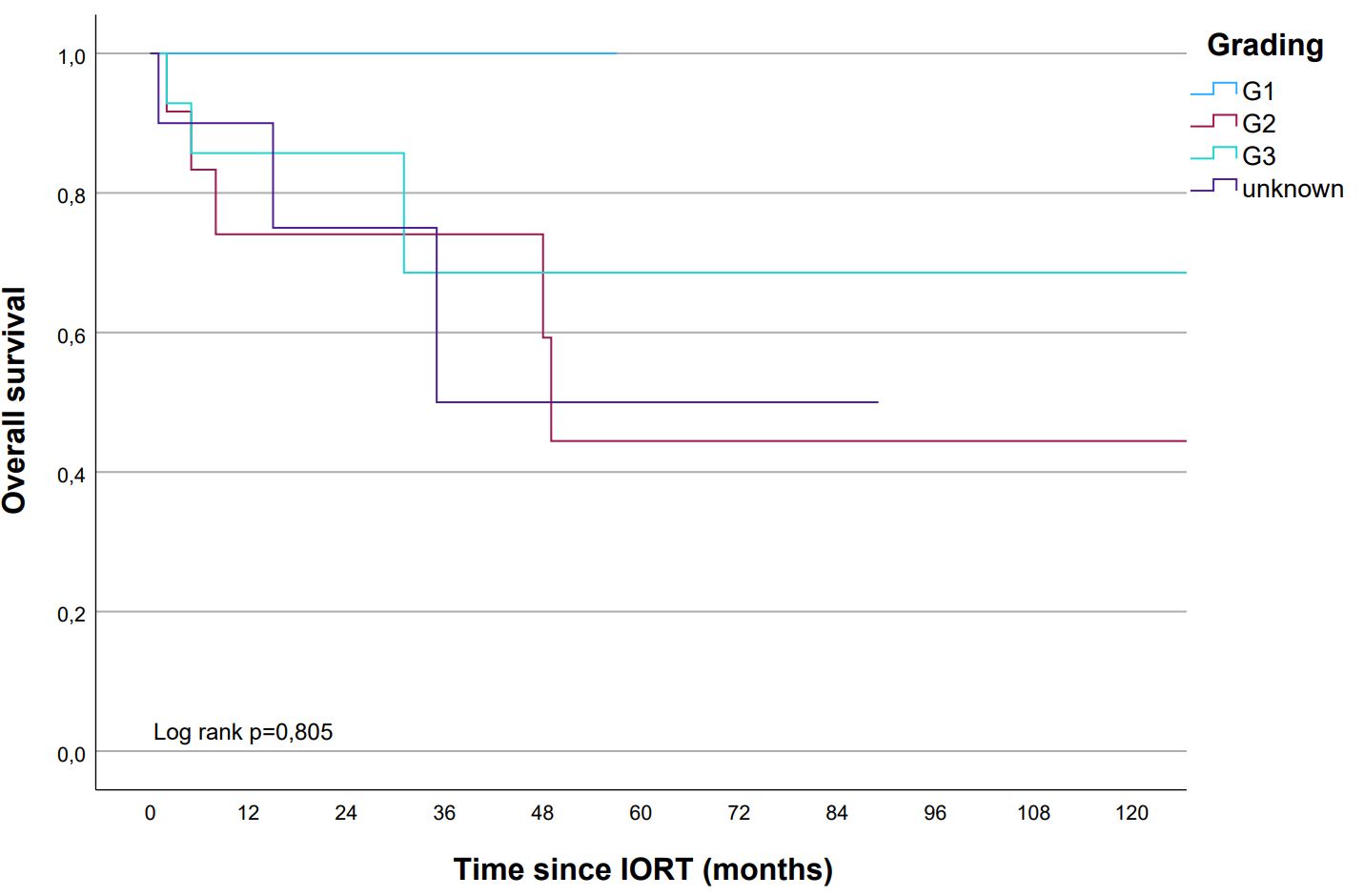

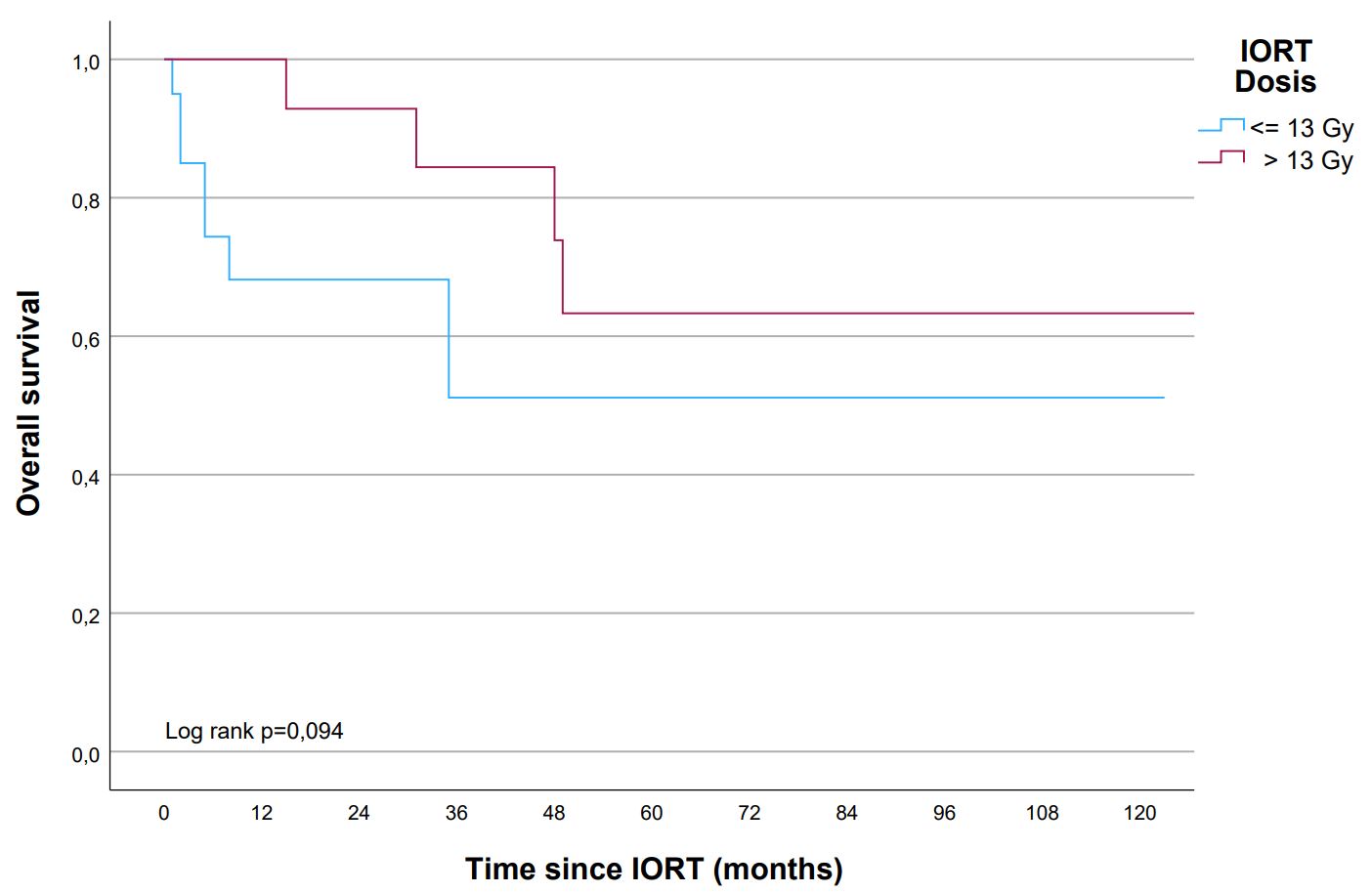


No at risk No at risk No at risk

Squamous 20 9 7 5 2 3 3 3 2 2 2 G1 1 1 1 1 1 1 ≤ 13 Gy 20 9 7 3 2 1 1 1 1 1 1

Adecoca. 9 8 6 4 4 2 2 2 2 2 2 G2 12 7 7 6 5 2 2 2 2 2 2 > 13 Gy 20 14 12 9 8 4 4 4 3 3 3

Other 11 6 6 3 2 G3 14 8 6 3 2 2 2 2 2 2 2

Unknown 13 10 5 2 2 1 1 1


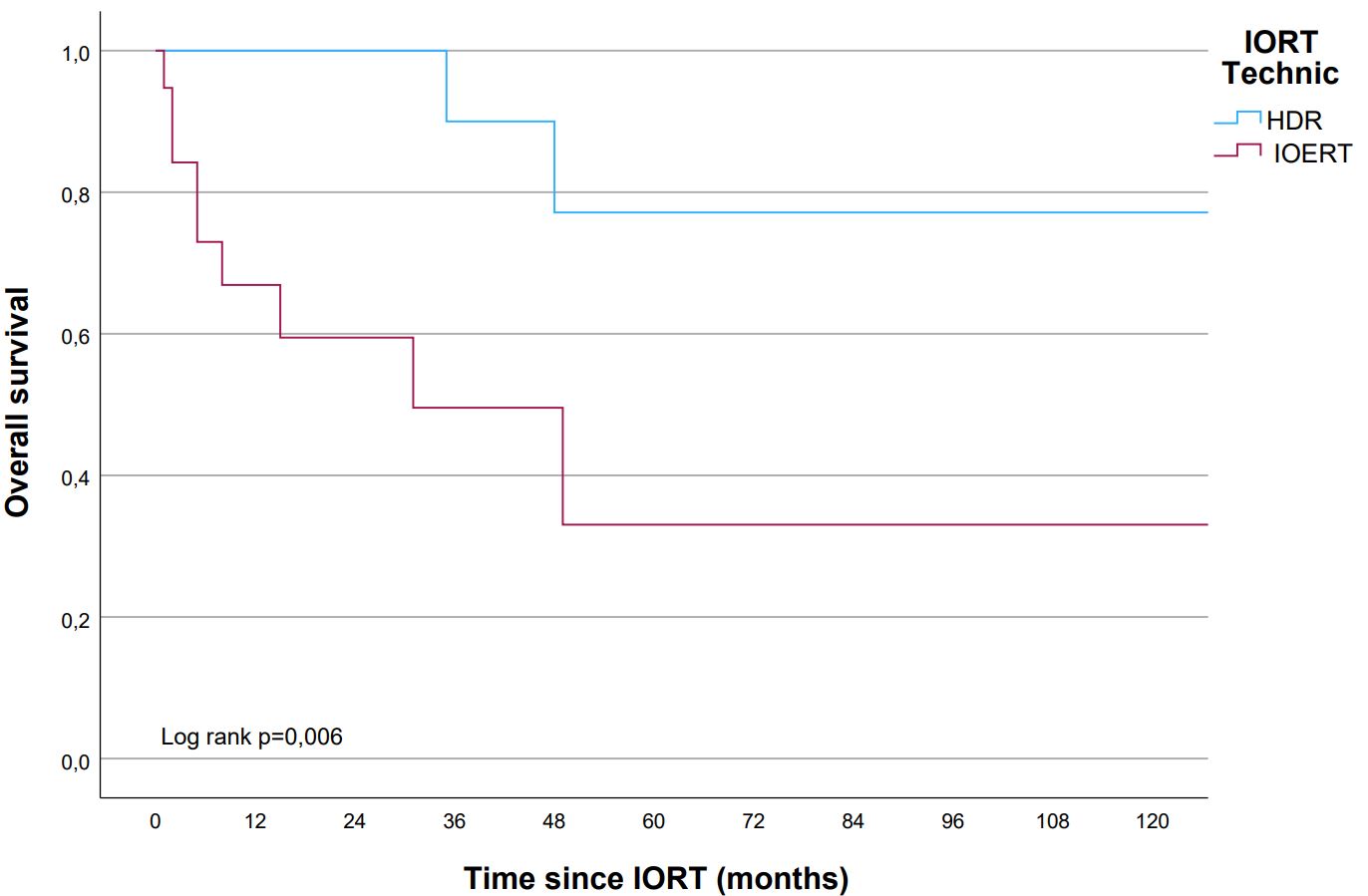

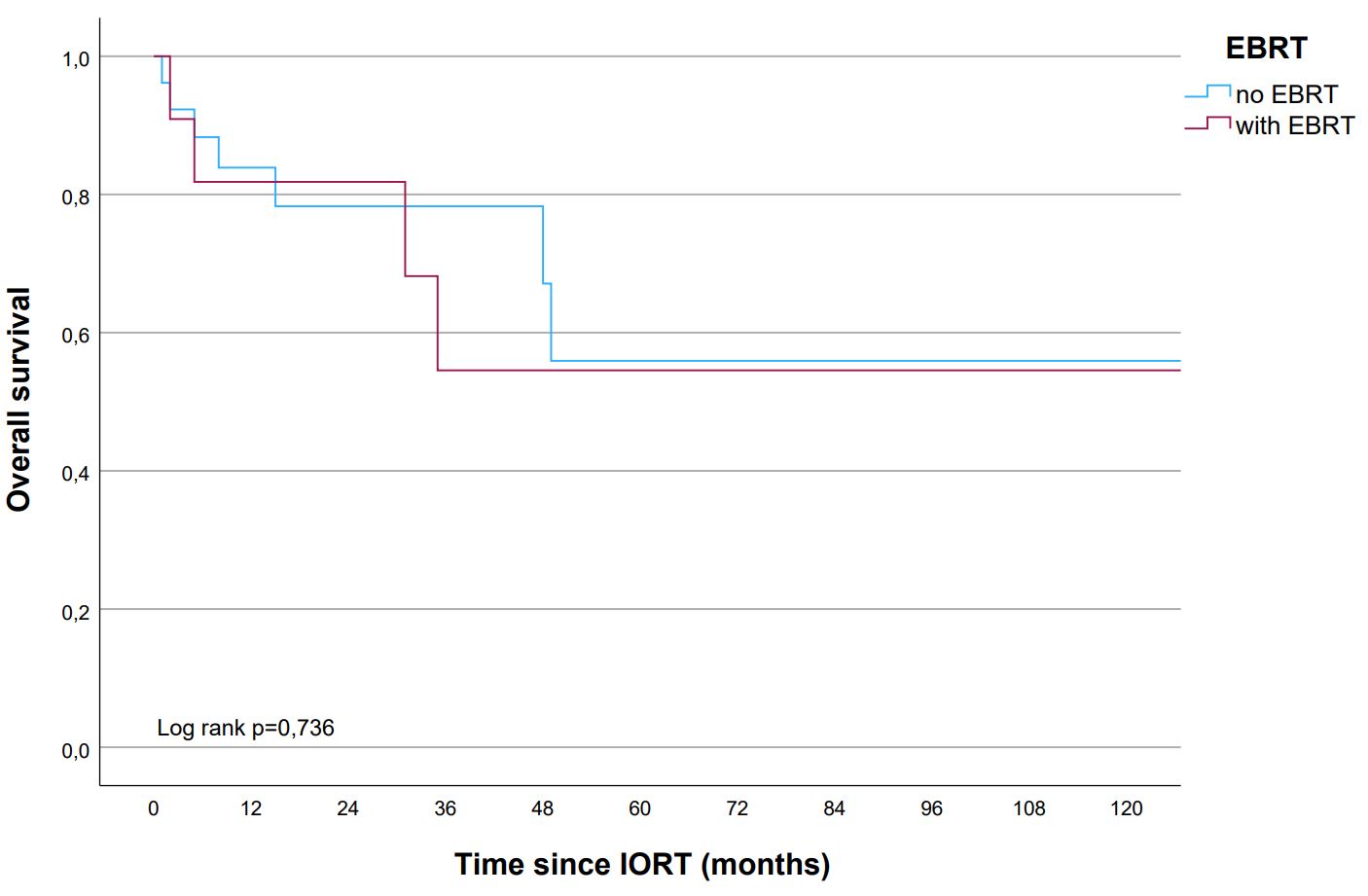


No at risk No at risk

HDR 21 11 9 4 2 with EBRT 11 7 7 4 3 2 2 2 2 2 2

IOERT 19 12 10 8 8 5 5 5 4 4 4 no EBRT 29 16 12 8 7 3 3 3 2 2 2

**Additional file 1: Figure S1:** Kaplan-Meier curves comparison for OS between groups classified by: Age at IORT and initial diagnosis (<65 vs. ≥65 years, respectively), disease-free interval (DFI) between initial diagnosis and first recurrence (<12 vs. ≥12 months), DFI to IORT (<12 vs. ≥12 months), grading, histology, adjuvant EBRT after IORT, IORT dose (≤13 vs. >13 Gy) and technique (high-dose radiotherapy (HDR) vs. IORT with electrons (IOERT)).
