# Supplementary material for: The value of a multimodal approach combining radical surgery and intraoperative radiotherapy in the recurrence treatment of gynecological malignancies - analysis of a large patient cohort in a tertiary care center
Source: Radiat Oncol. 2024 Oct 25;19:147. doi: 10.1186/s13014-024-02537-z (PMC11515090; doi:10.1186/s13014-024-02537-z)
Supplement: Supplementary file 2 — Supplementary Material 2 [file 13014_2024_2537_MOESM2_ESM.docx]

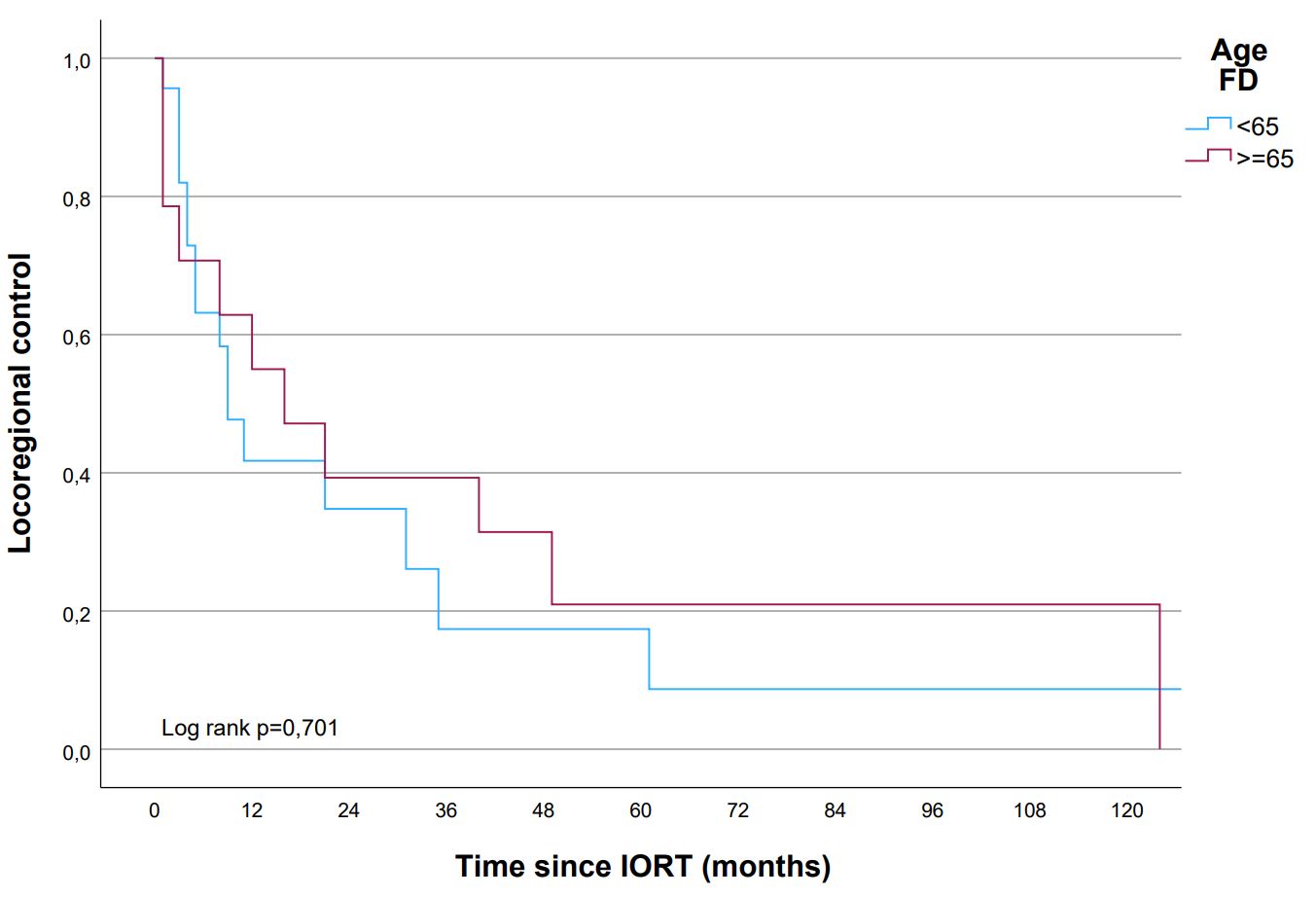

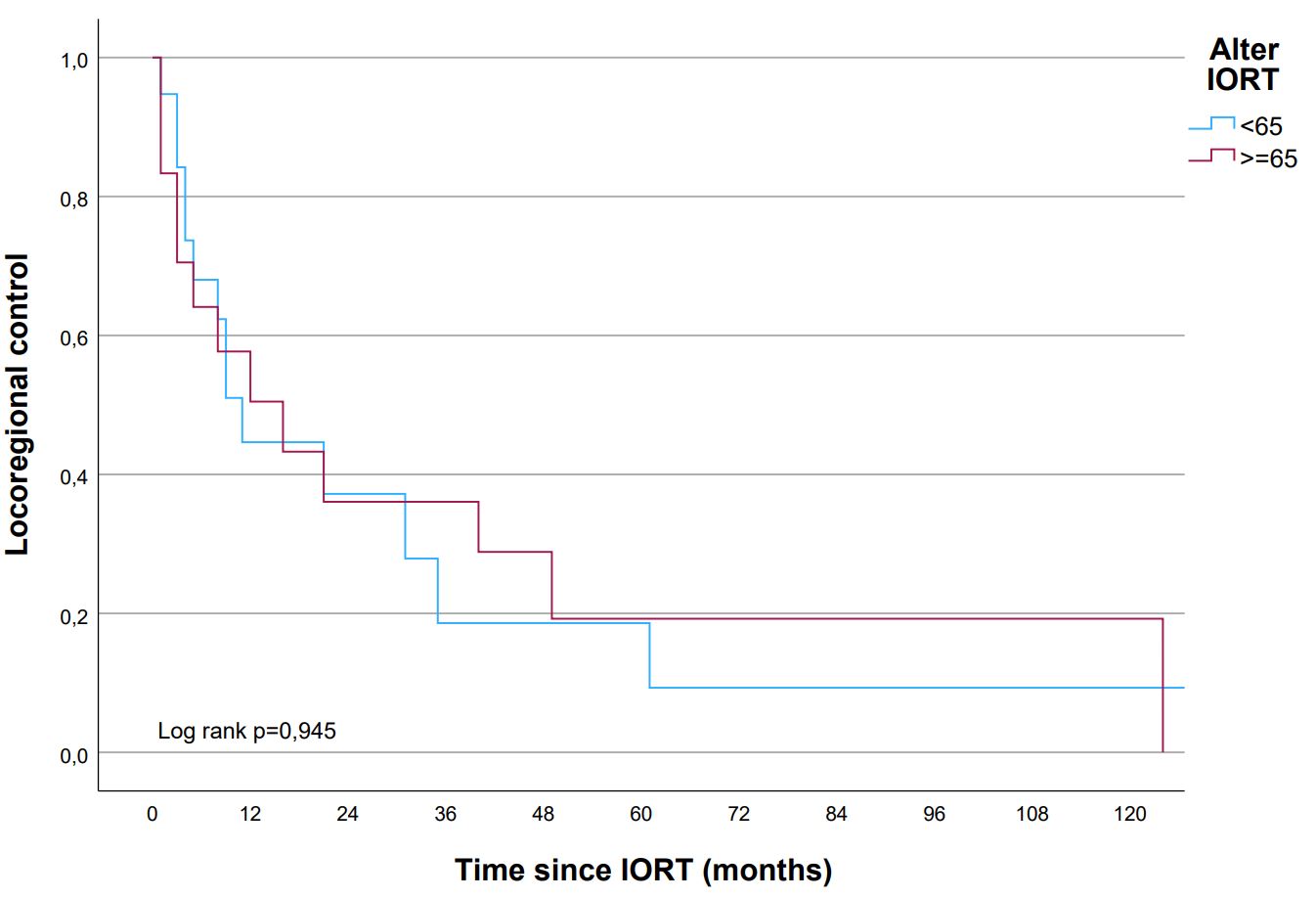

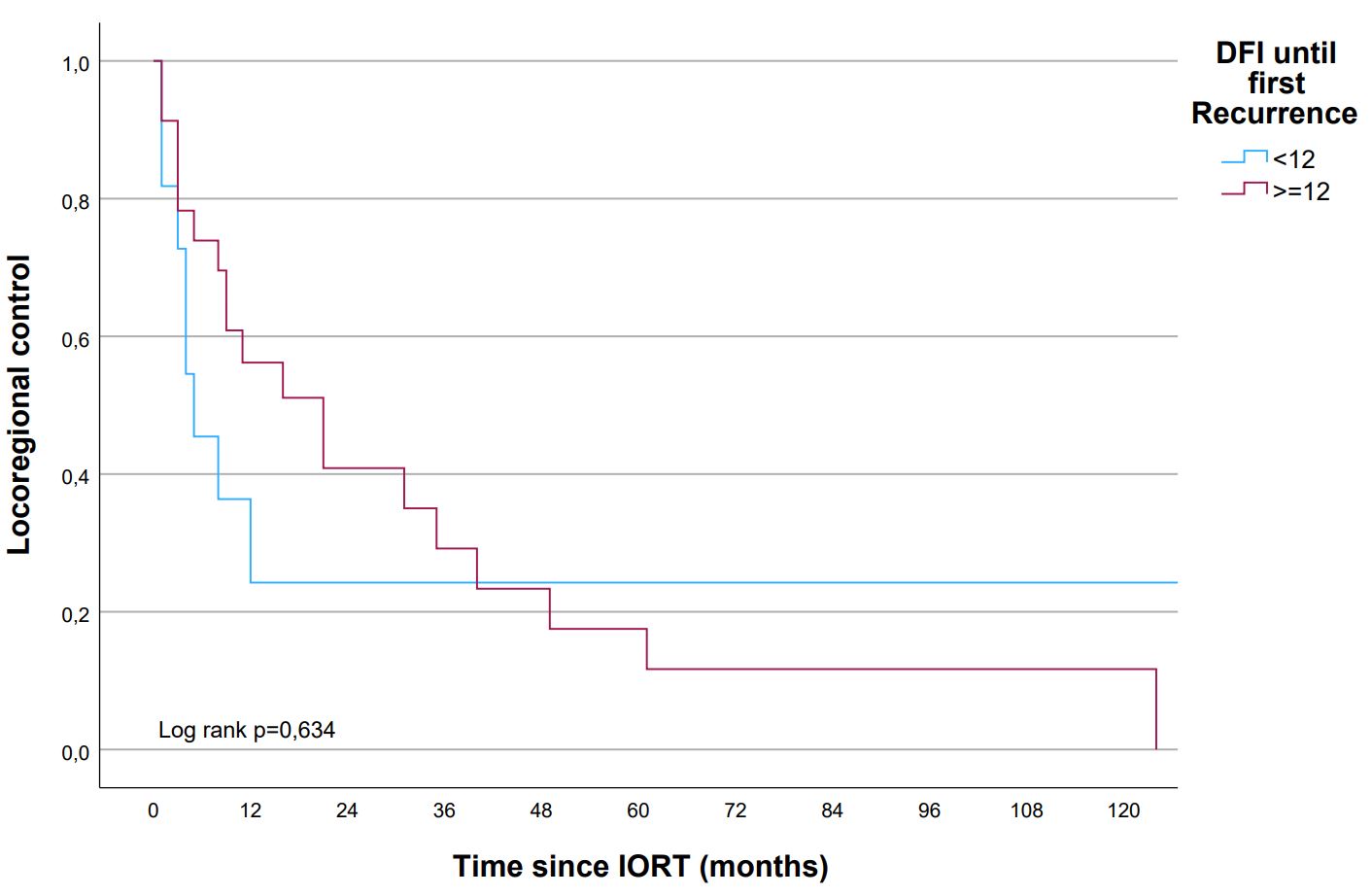

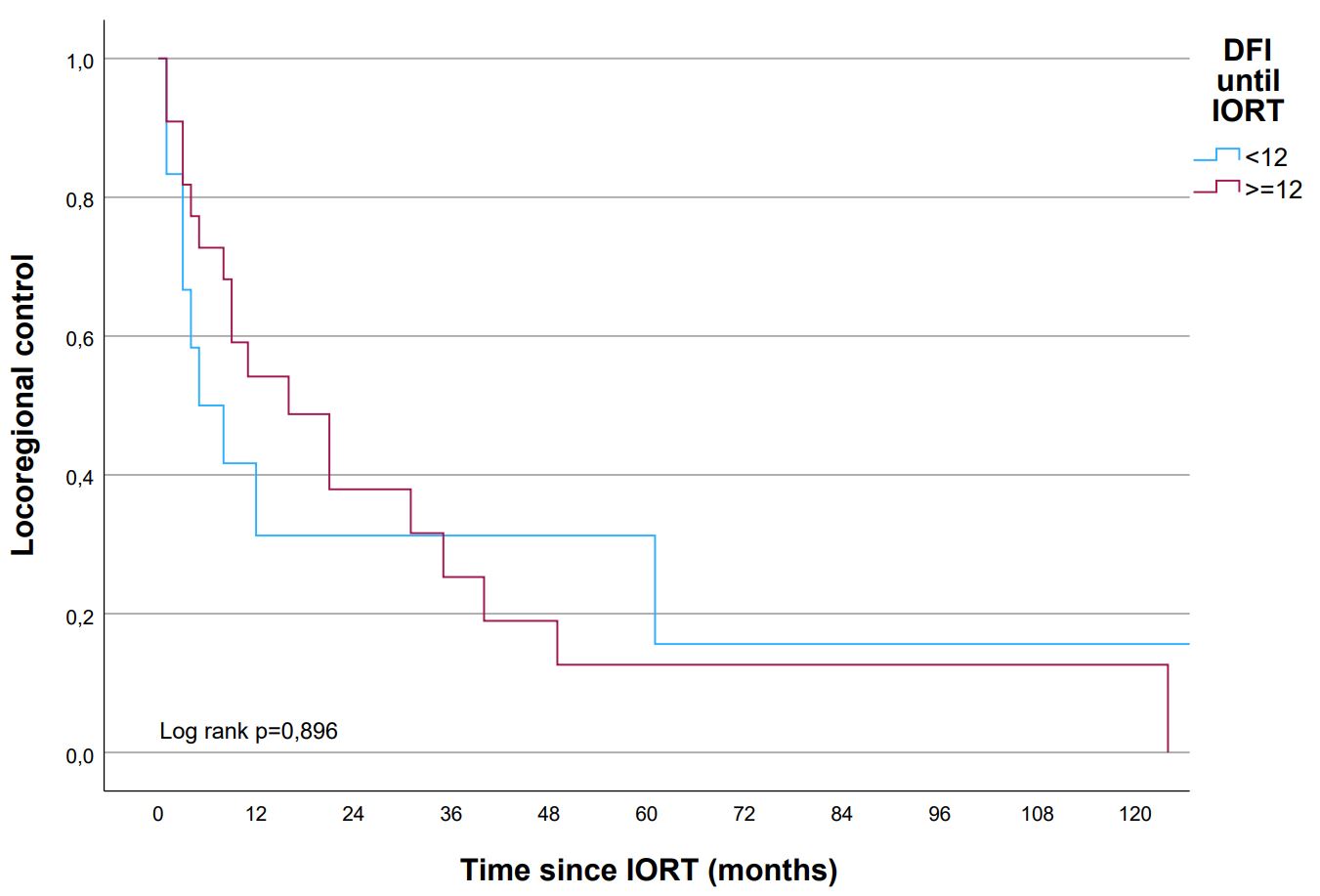


No at risk No at risk No at risk No at risk

<65 years 26 7 4 2 2 2 1 1 1 1 1 <65 years 22 7 4 2 2 2 1 1 1 1 1 <12 months 13 3 2 2 1 1 1 1 1 1 1 <12 months 13 4 3 3 2 2 1 1 1 1 1

≥65 years 14 8 5 5 3 2 2 2 2 2 2 ≥65 years 18 8 5 5 3 2 2 2 2 2 2 ≥12 months 24 17 15 9 8 3 3 3 2 2 2 ≥12 months 24 11 6 4 3 2 2 2 2 2 2


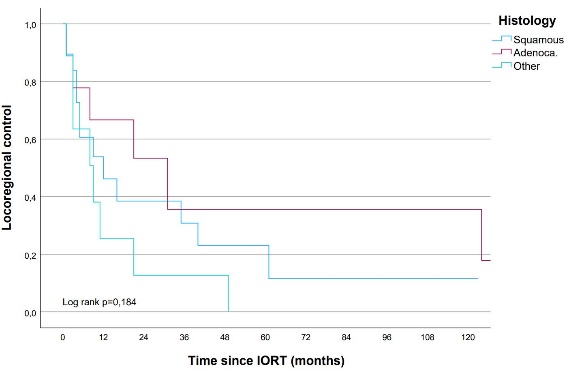

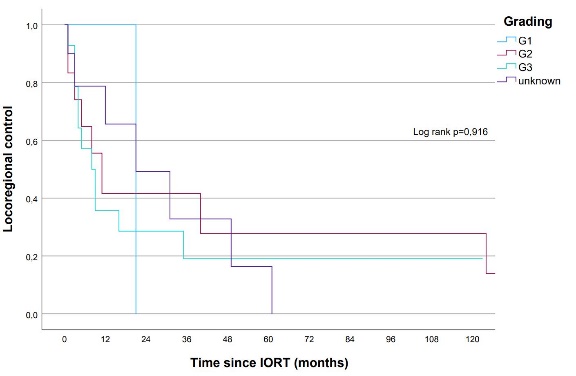


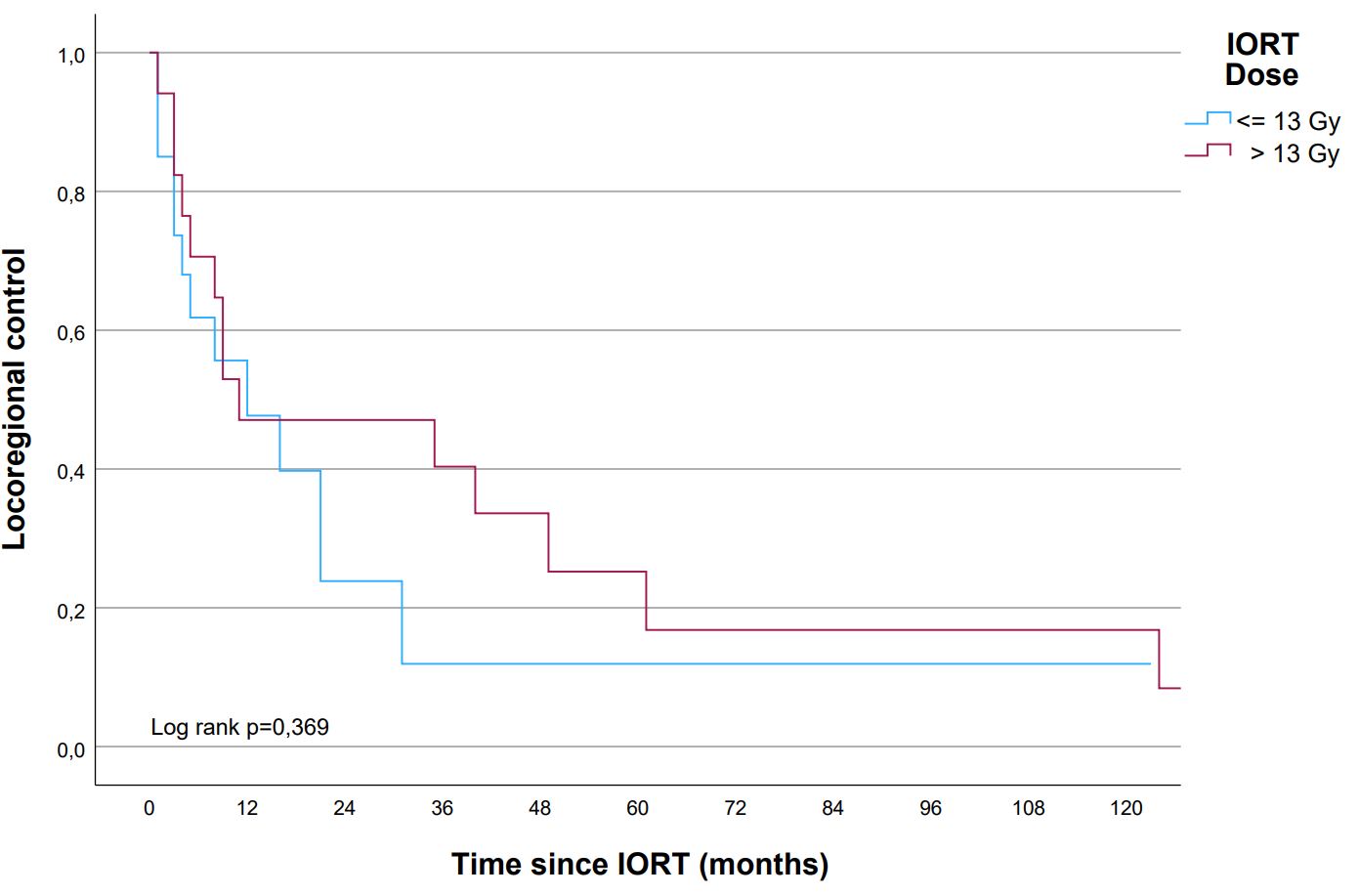


No at risk No at risk No at risk

Squamous 20 7 5 4 2 2 1 1 1 1 1 G1 1 1 1 1 1 1 ≤ 13 Gy 20 7 2 1 1 1 1 1 1 1 1

Adenoca. 9 6 3 2 2 2 2 2 2 2 2 G2 12 3 3 3 2 2 2 2 2 2 2 > 13 Gy 20 8 7 6 4 3 2 2 2 2 2

Other 11 2 1 1 1 G3 14 5 3 2 1 1 1 1 1 1 1

Unknown 13 6 3 2 2 1


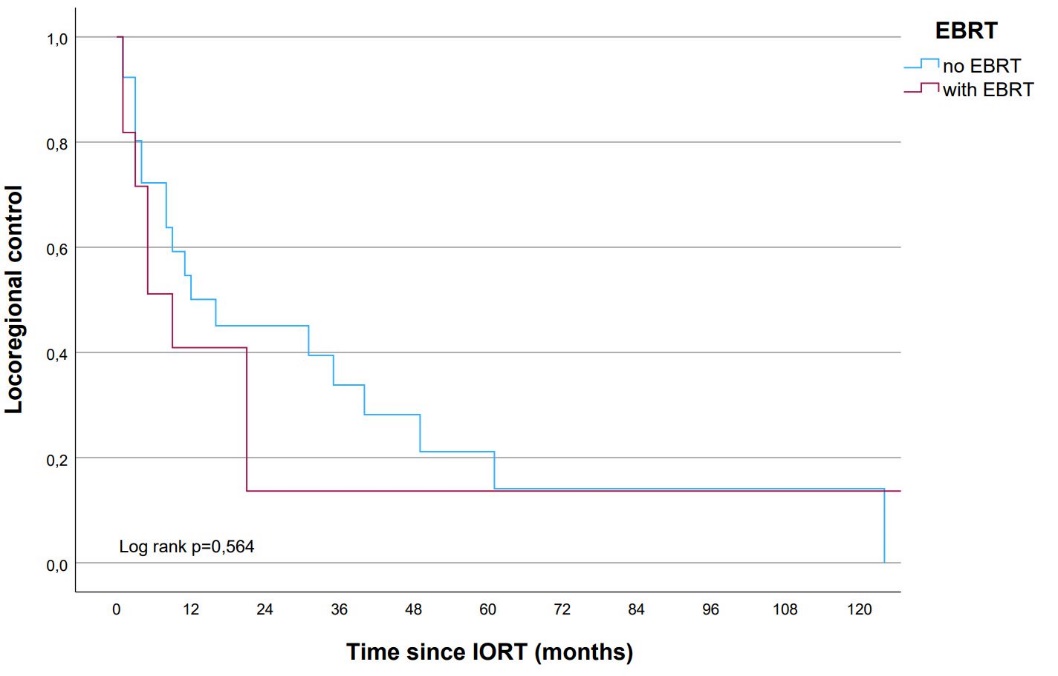


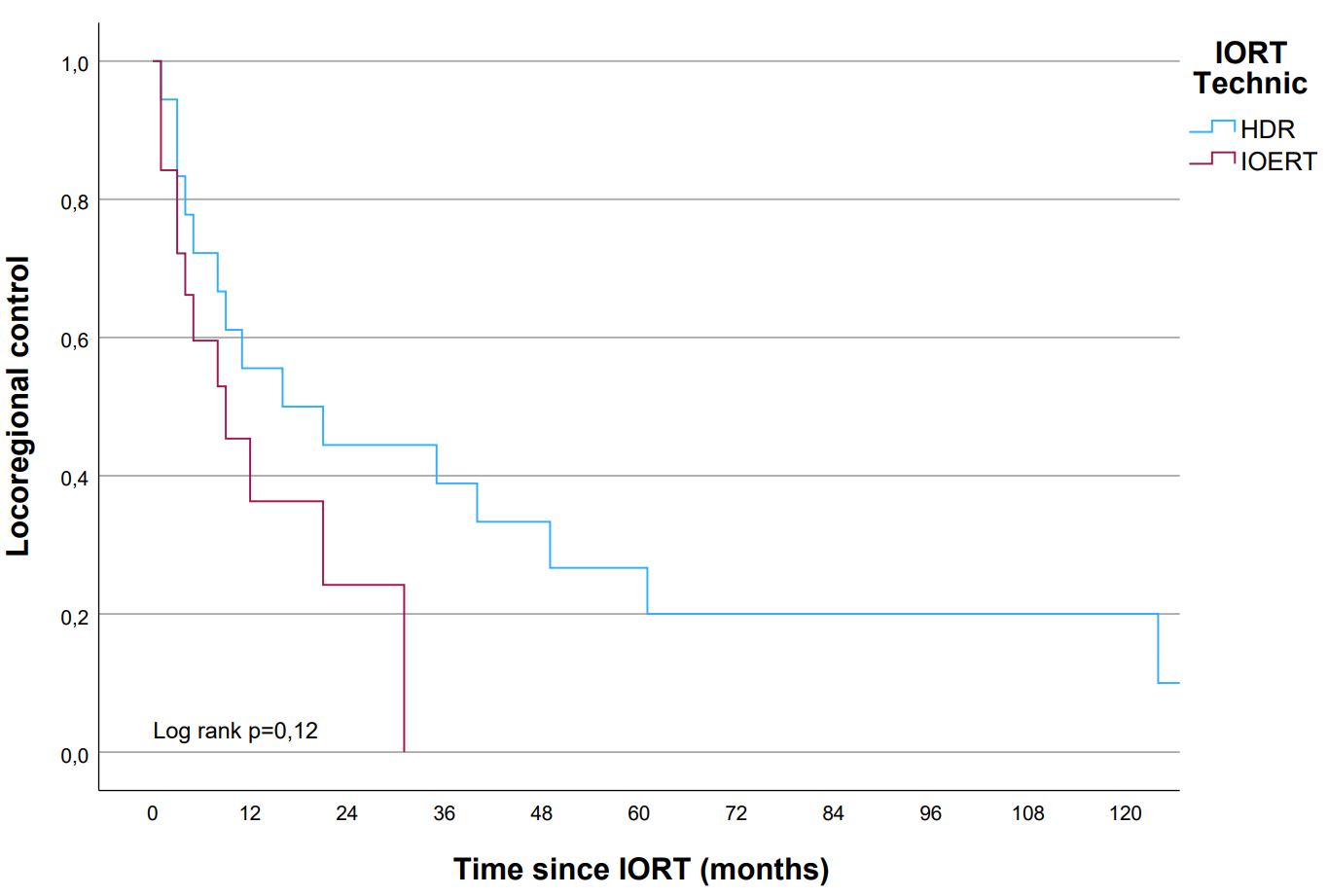


No at risk No at risk

HDR 21 10 8 7 5 4 3 3 3 3 3 with EBRT 11 3 1 1 1 1 1 1 1 1 1

IOERT 19 5 1 no EBRT 29 12 8 6 4 3 2 2 2 2 2

**Additional file 2: Figure S2:** Kaplan-Meier curves comparison for LRC between groups classified by: Age at IORT and initial diagnosis (<65 vs. ≥65 years, respectively), disease-free interval (DFI) between initial diagnosis and first recurrence (<12 vs. ≥12 months), DFI to IORT (<12 vs. ≥12 months), grading, histology, adjuvant EBRT after IORT, IORT dose (≤13 vs. >13 Gy) and technique (high-dose radiotherapy (HDR) vs. IORT with electrons (IOERT)).
