# Supplementary material for: The value of a multimodal approach combining radical surgery and intraoperative radiotherapy in the recurrence treatment of gynecological malignancies - analysis of a large patient cohort in a tertiary care center
Source: Radiat Oncol. 2024 Oct 25;19:147. doi: 10.1186/s13014-024-02537-z (PMC11515090; doi:10.1186/s13014-024-02537-z)
Supplement: Supplementary file 3 — Supplementary Material 3 [file 13014_2024_2537_MOESM3_ESM.docx]

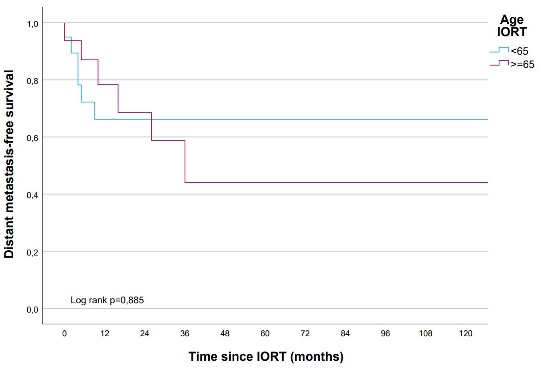

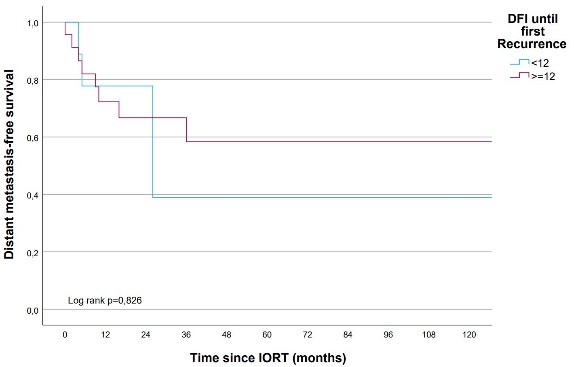


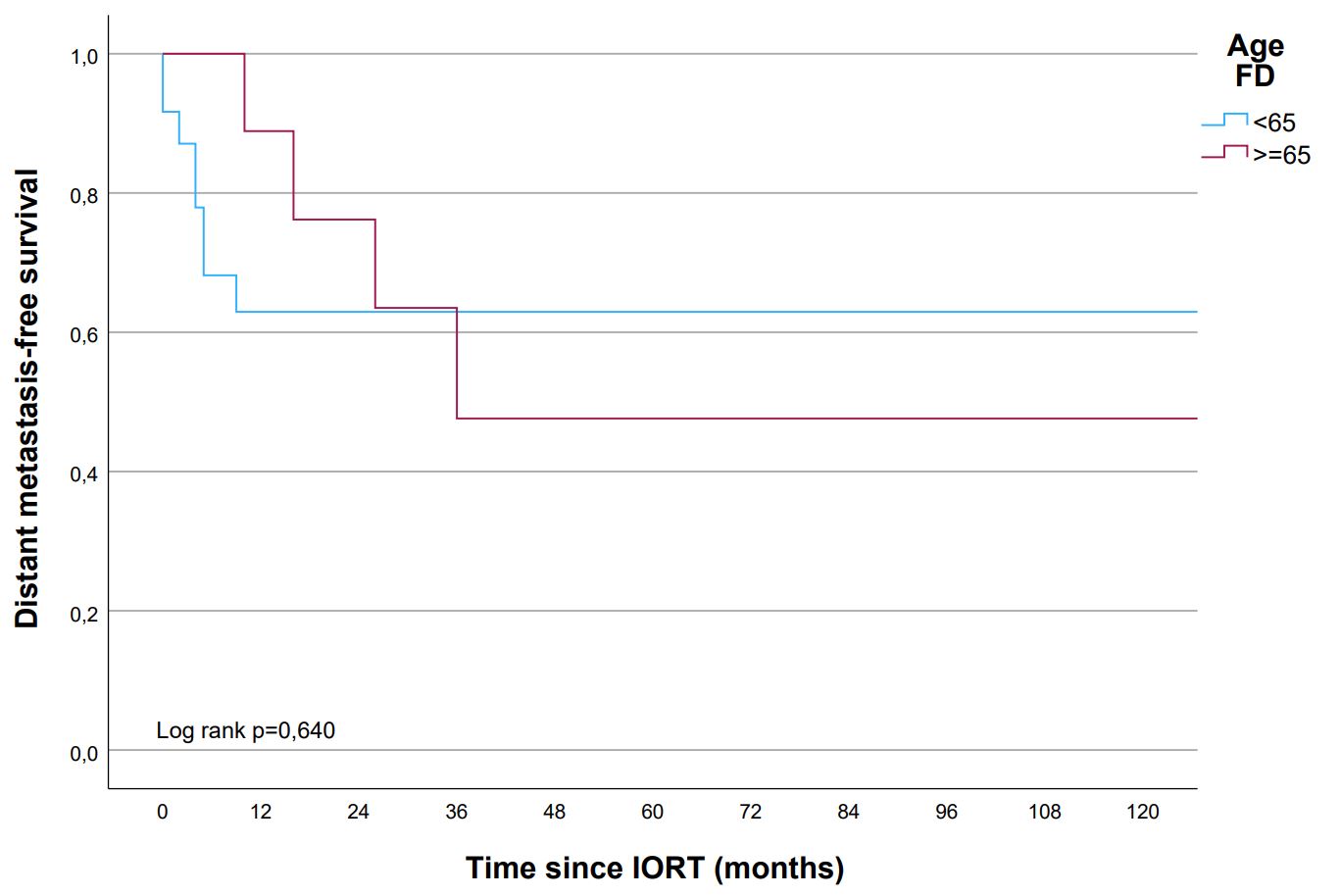

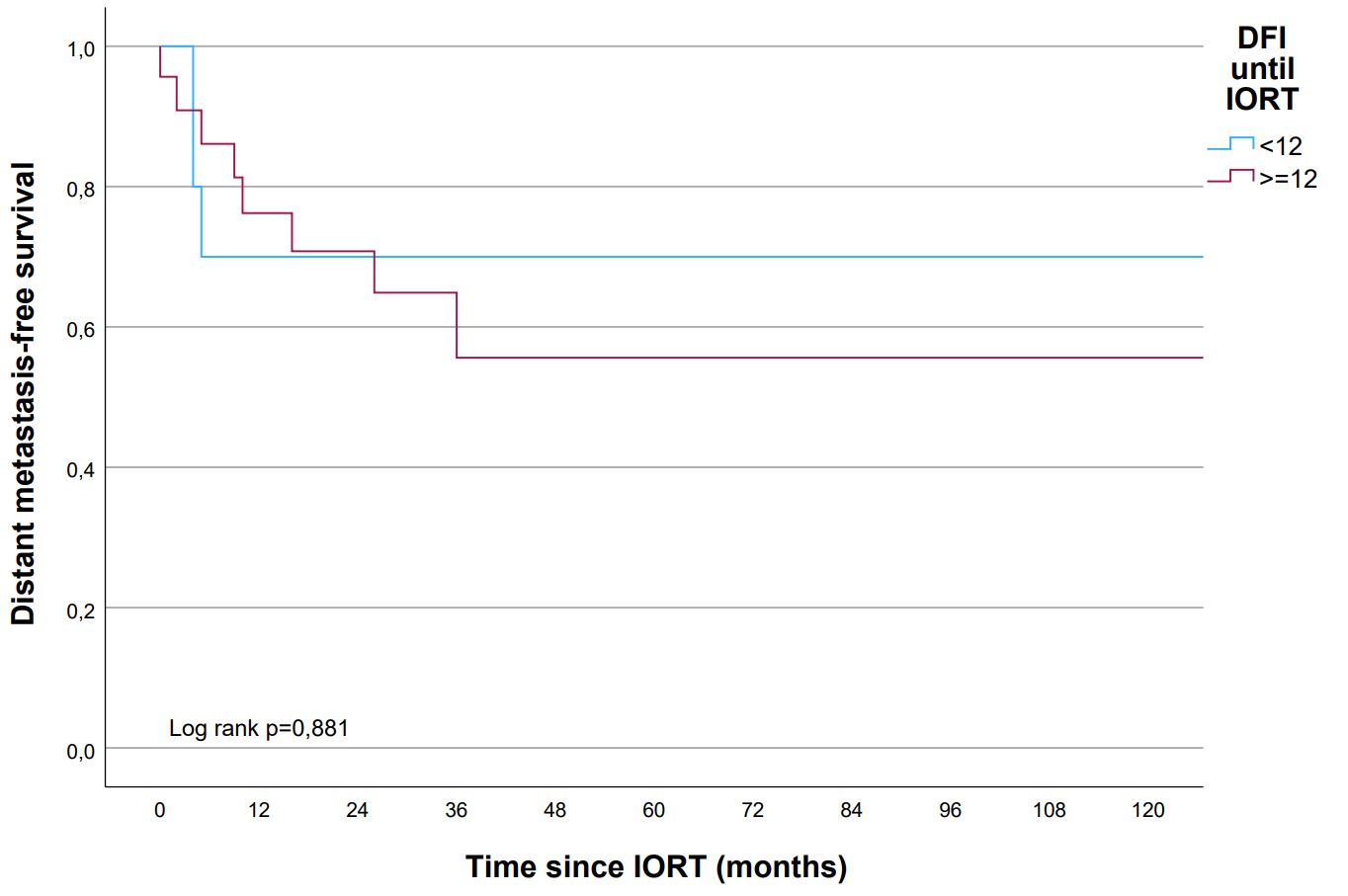


No at risk No at risk No at risk No at risk

<65 years 24 9 9 5 5 2 2 2 1 1 1 <65 years 24 9 8 5 5 2 2 2 1 1 1 <12 months 11 4 2 1 1 1 1 1 1 1 1 <12 months 11 3 2 2 2 2 2 2 1 1 1

≥65 years 12 8 5 4 3 2 2 2 2 2 2 ≥65 years 12 8 6 4 3 2 2 2 2 2 2 ≥12 months 23 13 13 8 7 3 3 3 2 2 2 ≥12 months 23 14 12 7 6 2 2 2 2 2 2


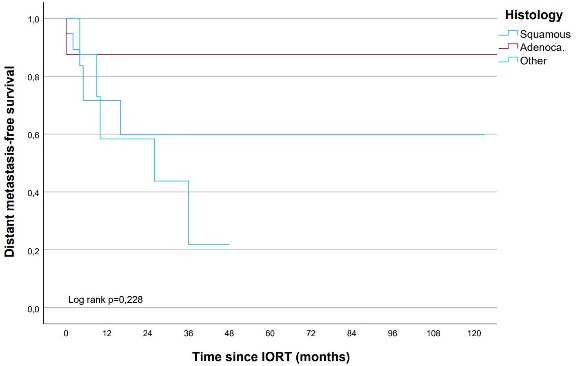

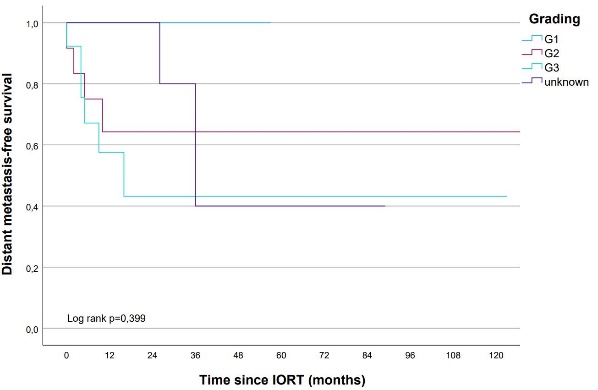


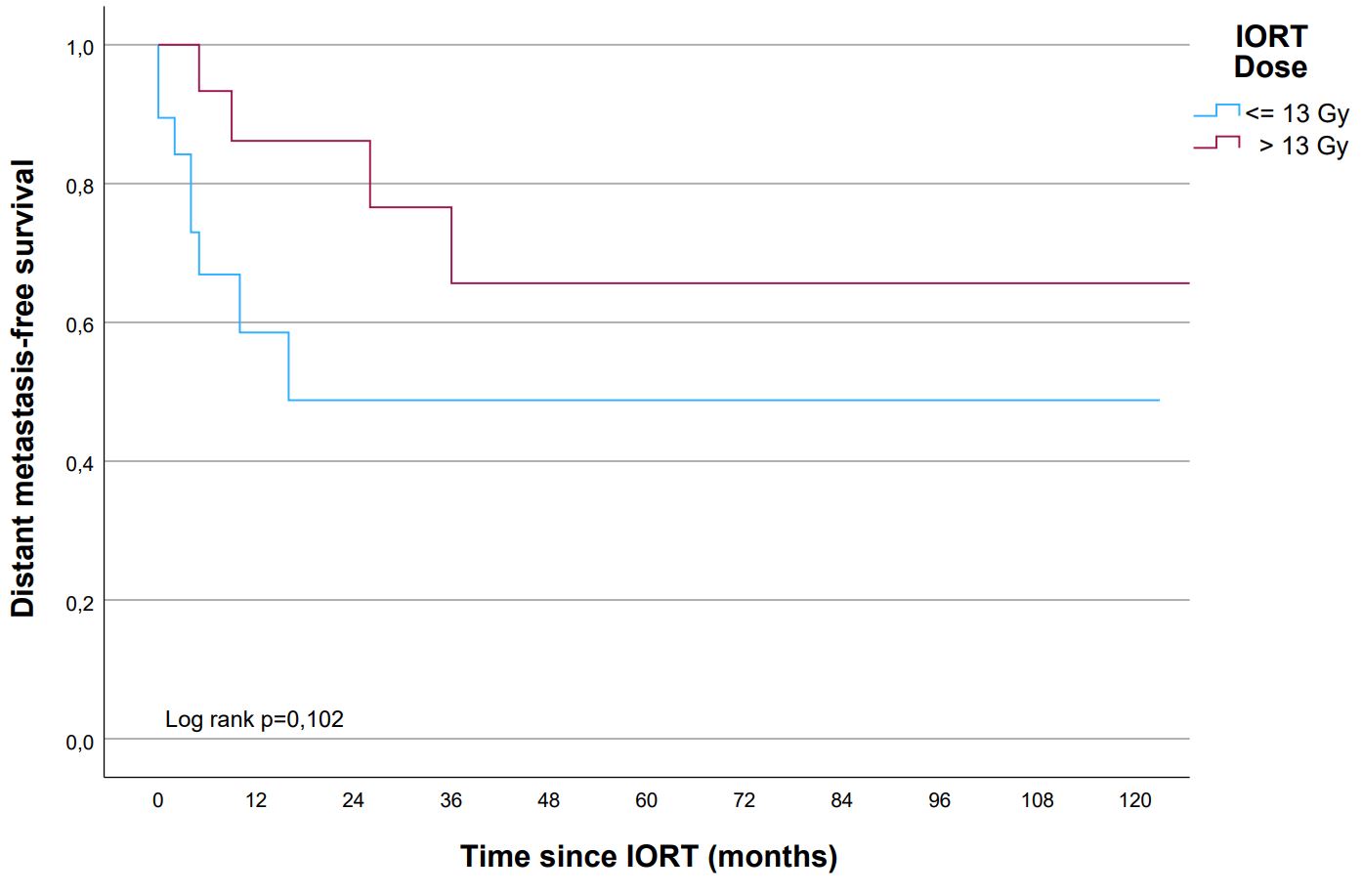


No at risk No at risk No at risk

Squamous 19 7 4 3 3 2 2 2 1 1 1 G1 1 1 1 1 1 ≤ 13 Gy 19 7 5 2 2 1 1 1 1 1 1

Adenoca. 8 6 6 4 4 2 2 2 2 2 2 G2 12 6 6 5 5 2 2 2 2 2 2 > 13 Gy 17 10 9 7 6 3 3 3 2 2 2

Other 9 4 4 2 1 G3 13 4 2 1 1 1 1 1 1 1 1

Unknown 10 6 5 2 1 1 1 1


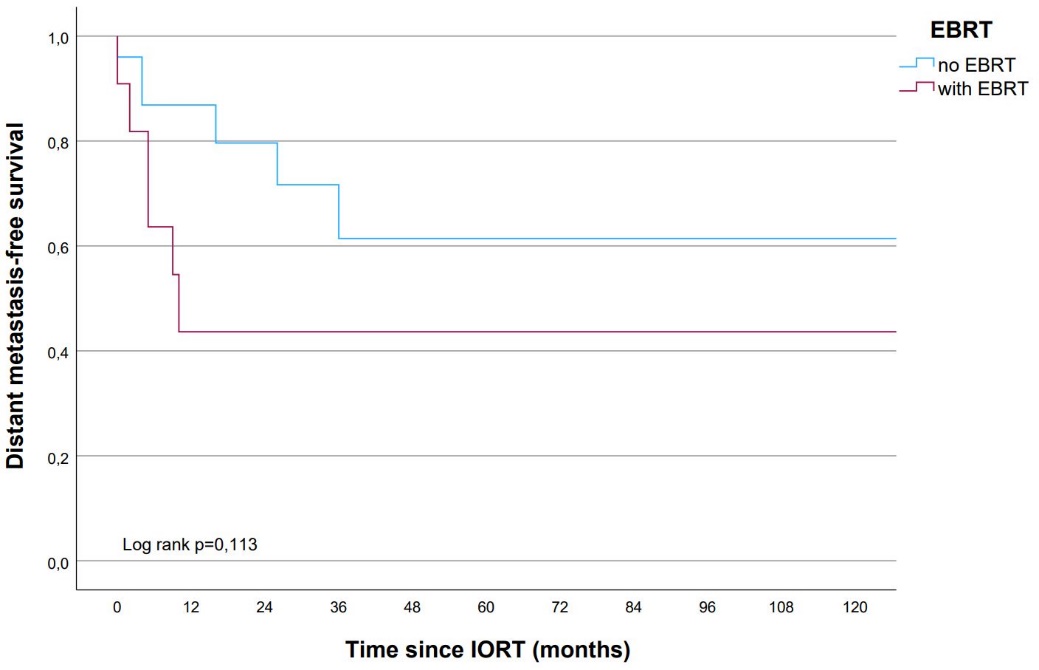


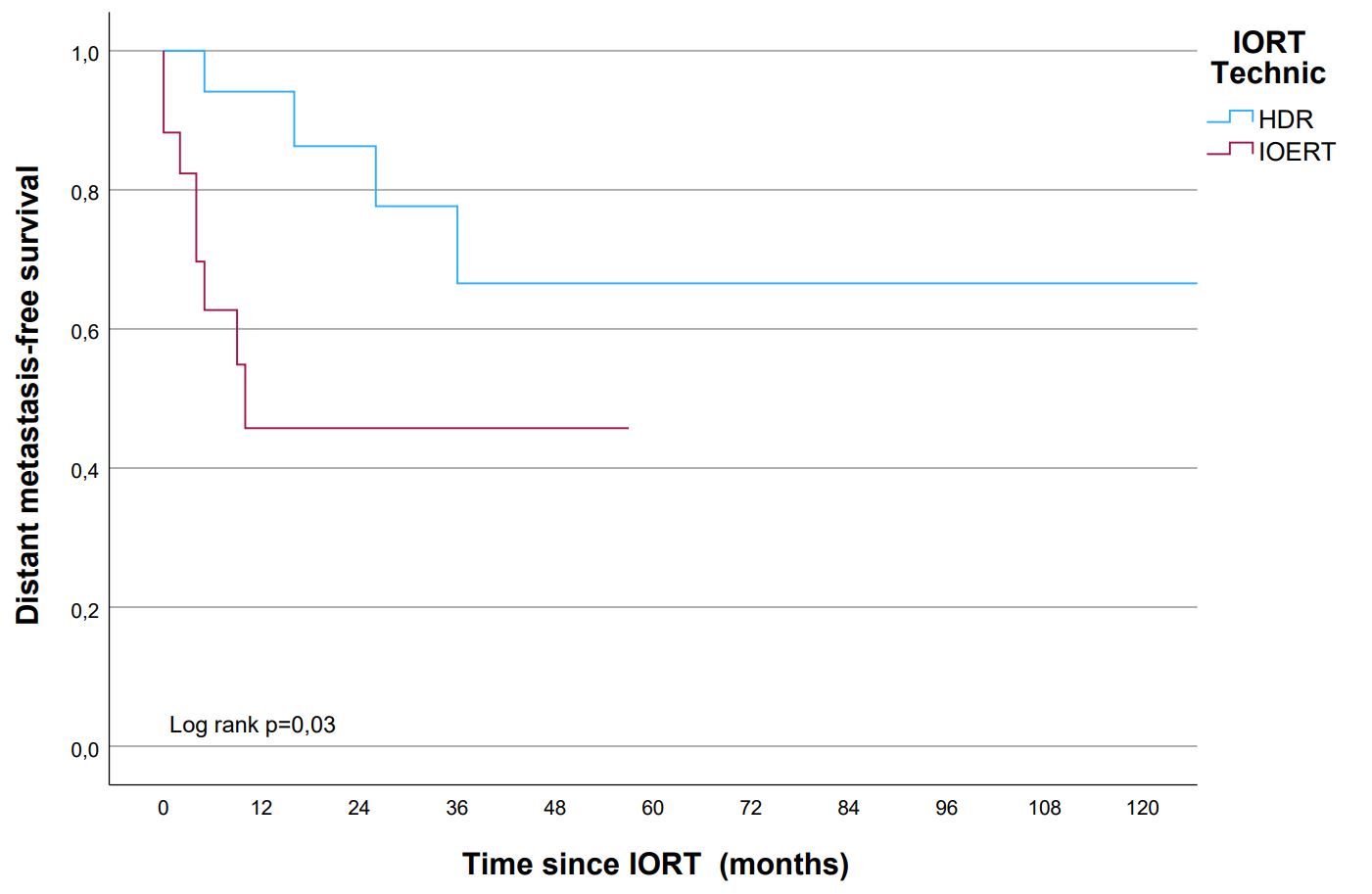


No at risk No at risk

HDR 19 12 10 7 6 4 4 4 3 3 3 no EBRT 25 13 10 7 6 3 3 3 2 2 2

IOERT 17 5 4 2 2 with EBRT 11 4 4 2 2 1 1 1 1 1 1

**Additional file 3: Figure S3:** Kaplan-Meier curves comparison for DMFS between groups classified by: Age at IORT and initial diagnosis (<65 vs. ≥65 years, respectively), disease-free interval (DFI) between initial diagnosis and first recurrence (<12 vs. ≥12 months), DFI to IORT (<12 vs. ≥12 months), grading, histology, adjuvant EBRT after IORT, IORT dose (≤13 vs. >13 Gy) and technique (high-dose radiotherapy (HDR) vs. IORT with electrons (IOERT)).
